# Reconstruction Assisted Semi-Supervised Conformer for Bolt Looseness Diagnosis in Large-Scale Bolted Mechanical Structures Under Heterogeneous Acquisition Conditions

**DOI:** 10.3390/s26195998

**Published:** 2026-09-22

**Authors:** Jinghao Cao, Jiaxing Liu, Hairong Wang, Shijie Su

**Affiliations:** 1 School of Mechanical Engineering, Jiangsu University of Science and Technology, Zhenjiang 212100, China; cjh@just.edu.cn (J.C.); 261210201513@stu.just.edu.cn (J.L.); 2 Zhoushan Institute of Calibration and Testing for Quality and Technology Supervision, Zhoushan 316021, China; 231110201111@stu.just.edu.cn

**Keywords:** bolt looseness diagnosis, vibration signal, MFCC, semi-supervised learning, self-supervised reconstruction, conformer, structural health monitoring

## Abstract

**Highlights:**

**What are the main findings?**

- Reconstruction-assisted learning under a grouped protocol: Labeled samples supervise bolt-state classification, while labeled and unlabeled MFCCs support denoising and masked reconstruction without state pseudo-labels.
- Grouped performance with explicit uncertainty: Across five seeds, the full model reached test macro-F1 0.9124 ± 0.0033, while held-out tower types, a later cohort, and waveform disturbances revealed substantial transfer limitations.

**What are the implications of the main findings?**

- Qualified evidence for unlabeled reconstruction: The mean gain over labeled reconstruction was small and its seed-level 95% interval included zero; reduced-label experiments showed no consistent annotation-saving benefit.
- Defines the present deployment limits: Validation-only calibration, class-wise metrics, alert-burden scenarios, and waveform stress tests show that prospective tower-separated and field-noise validation remain necessary.

**Abstract:**

Bolt looseness diagnosis in power transmission towers is complicated by costly state labels and changing acquisition conditions. This study evaluates a reconstruction-assisted semi-supervised conformer using triaxial vibration-derived MFCCs. Labeled samples supervise binary classification, while labeled and unlabeled samples support denoising and masked reconstruction without state pseudo-labels. A retrospective audit retains 70,409 labeled and 154,394 distinct unlabeled segments and separates 42,421 training, 13,934 validation, and 14,054 test segments by identifiable source-record, inferred-acquisition, and exact-feature groups. Across five seeds, the full model obtains test macro-F1 0.9124 ± 0.0033 and ROC–AUC 0.9692 ± 0.0025, compared with 0.9032 ± 0.0135 and 0.9644 ± 0.0074 for labeled reconstruction. The paired macro-F1 difference is 0.00915 with a 95% seed interval of −0.00503 to 0.02333; reduced-label experiments show no consistent benefit. Performance is substantially weaker on held-out tower types and a later cohort, and waveform-level perturbations cause marked degradation. At clean inference, one encoder call exactly preserves the original duplicated-feature predictions. The results support useful grouped within-archive classification but do not establish physical-tower-independent generalization, annotation savings, or validated field-noise tolerance.

## 1. Introduction

Transmission towers are safety-critical load-bearing components of electric power networks. Their structural condition directly affects the continuity and reliability of power delivery. Bolted joints are extensively used to connect tower members and remain exposed throughout service to wind loading, conductor-induced vibration, temperature variation, and other environmental actions [1]. Once a bolt loses preload, the contact stiffness, damping, and vibration-transmission characteristics of the joint can change. If such degradation is not detected and corrected in time, damage may accumulate under cyclic loading, progressively impair the integrity of the connection, and, in severe cases, contribute to tower instability or collapse. The resulting consequences may include service interruption, equipment damage, substantial economic loss, and risks to personnel. Timely and reliable assessment of bolted connections is therefore an important part of transmission-infrastructure maintenance [2,3,4,5,6,7,8,9,10,11,12].

Bolted connections in lattice transmission towers are exposed to repeated transverse loading, conductor-induced vibration, wind excitation, temperature variation, and repeated maintenance operations. Loss of bolt preload changes the contact conditions and may reduce the connection’s resistance to further movement [13]. Quantitative defect records illustrate the maintenance relevance of this problem, although their denominators require careful interpretation. Li et al. [14] summarized power-management-system records collected from January 2015 to April 2020, in which loose-bolt entries represented 7.2–19.1% of recorded tower defects across ten provincial datasets. In the Shanxi dataset, 2193 of 29,492 recorded tower defects were classified as bolt loosening, corresponding to 7.4%. These values are historical proportions among reported defects rather than estimates of the prevalence of loose bolts among all installed bolts, annual incidence rates, or current national prevalence.

More recent studies have examined the loosening process of transmission-tower bolted connections under transverse cyclic loading [15] and vibration-based monitoring under different excitation and sensor arrangements [16]. These studies show that bolt loosening is associated with repeated loading, loss of preload, changes in contact conditions, and variations in joint stiffness and vibration transmission. They also show that a measured response can depend on excitation position, sensor position, tower geometry, and the vibration-propagation path.

Existing maintenance approaches include visual inspection, verification of tightening condition using torque tools, and anti-loosening components appropriate to the connection design. These measures are necessary for prevention and confirmation but require physical access to the relevant joint, and visual inspection may not identify incipient preload loss. Vibration, acoustic, impedance, guided-wave, optical, and magnetic methods provide complementary diagnostic information, but they can depend on dedicated instrumentation, sensor coupling, excitation repeatability, propagation path, and structure-specific signal processing. These limitations motivate the investigation of a vibration-based machine-learning classifier as a screening tool. Its output should not be interpreted as a direct measurement of bolt preload or as a replacement for engineering confirmation of an alarm.

Despite encouraging results, a substantial gap remains between many existing demonstrations and the requirements of field-scale transmission-tower inspection. Some studies [17,18,19,20] have been conducted on small laboratory joints, a single specimen, or highly controlled acquisition setups in which the structural configuration, excitation procedure, and sensor arrangement are similar across training and evaluation data. Such results do not by themselves establish reliability under the heterogeneous conditions encountered on full-scale towers. Other studies have focused on one tower design or a fixed impact–sensor geometry. Because tower topology, propagation path, and acquisition geometry all affect the measured response, high accuracy under a single configuration does not necessarily imply stable performance across tower types or alternative measurement arrangements.

Our earlier supervised work [20] investigated bolt-looseness recognition using multiple signal modalities and showed that supervised models could distinguish bolt conditions in data collected from multiple tower types represented in the dataset. However, supervised learning depends on a sufficiently large, accurately labeled, and condition-diverse training set. Changes in tower type, impact location, sensor location, or propagation path can shift the input distribution and encourage a classifier to exploit configuration-specific patterns rather than state-related features that transfer across conditions. More importantly, reliable labels require the physical bolt state to be deliberately established and verified, making labeled data substantially more expensive and difficult to acquire than unlabeled vibration records.

This labeling difficulty is not merely a conventional annotation burden; it follows directly from the engineering procedure used to construct bolt-condition data. On an experimental tower, many loose configurations can be generated by changing the locations, numbers, and combinations of loosened bolts and by repeatedly acquiring responses under each configuration. A fully tightened tower, in contrast, usually corresponds to one baseline connection configuration. Repeated measurements can increase the number of segments, but the number of physically distinct fully tightened configurations remains much smaller than the number of loose configurations that can be created combinatorially. Additional tight configurations require retightening, preload verification, and rechecking of the acquisition setup, all of which increase experimental effort.

The asymmetry becomes more pronounced on in-service towers. Vibration responses can be collected without intentionally changing structural conditions, making it feasible to accumulate unlabeled responses under a broader range of operating and acquisition conditions. Obtaining confirmed loose-state labels, however, may require bolts to be loosened, dismantled, or individually verified. Such intervention introduces additional work at height, site coordination, and safety concerns and cannot be repeated extensively on operating infrastructure. The resulting data regime is therefore asymmetric in two ways: experimental campaigns can produce many loose-state examples but relatively few distinct tight-state configurations, while field campaigns can produce diverse unlabeled responses but only a limited number of reliably confirmed state labels.

After the reproducibility audit, the study retains 70,409 labeled MFCC segments and 154,394 distinct unlabeled segments. The labeled archive is assigned grouped training, validation, and test roles; unlabeled samples contribute reconstruction losses only and never receive a state pseudo-label.

This study addresses the following question: can a large pool of unlabeled vibration data be combined with a smaller, imbalanced labeled set with limited condition coverage to learn a more useful diagnostic representation than supervised classification alone? Instead of treating each unlabeled segment as a sample with an unobserved class label that must be estimated, we use the MFCC itself to define denoising and masked-reconstruction objectives. Labeled samples supervise the diagnostic boundary, whereas labeled and unlabeled samples jointly constrain the encoder to recover structure in the implemented MFCC token–feature array. This division of training roles allows unlabeled data to contribute to representation learning without requiring an assumed bolt-state label.

As shown in Figure 1, to implement this idea, we develop a reconstruction-assisted semi-supervised conformer network for transmission-tower bolt looseness diagnosis. The MFCC is represented as a temporal token–feature array. Multi-head self-attention models dependencies across axis-specific temporal tokens, while the convolutional module captures local adjacency within the implemented token sequence. Token-level residual denoising and contiguous token-axis and feature-axis masking require the shared encoder to reconstruct the corresponding pre-corruption MFCC array from perturbed views. Corrupted-input classification, uncorrupted-input classification, and reconstruction objectives over the labeled and unlabeled pools are optimized jointly.

The revised evaluation separates validation-based model selection and calibration from final test inference. It further uses paired five-seed comparisons, controlled component ablations, reduced-label experiments, held-out tower-type tests, a later acquisition cohort, and waveform-level perturbations. These analyses distinguish favorable within-archive performance from transfer and deployment limitations.

The main contributions of this work are as follows:

1. We formulate the asymmetric-supervision problem from the engineering process of transmission-tower data acquisition. The ability to create many loose configurations, the limited diversity of fully tightened configurations, and the difficulty of manipulating in-service structures jointly lead to severe class imbalance, incomplete condition coverage, and a large pool of unused unlabeled responses.
2. We introduce a task-partitioned semi-supervised strategy in which labeled samples learn the tight/loose decision boundary, while both labeled and unlabeled MFCCs supervise denoising and masked reconstruction. The unlabeled pool is therefore exploited without assigning potentially unreliable class pseudo-labels.
3. We develop a conformer-based diagnostic model that combines attention across token positions, convolution over local adjacency in the implemented token order, token-level residual denoising, and contiguous-region reconstruction within a shared encoder.
4. We use grouped training, validation, and test roles and conduct paired multi-seed comparisons, controlled ablations, reduced-label experiments, tower-type transfer tests, probability calibration, and waveform stress tests. The revised evidence supports within-archive classification but not universal gains from unlabeled reconstruction or tower-independent deployment.

Accordingly, the purpose of this work is not merely to report a high classification score on the available data. It is to investigate how labeled and unlabeled vibration records can be used responsibly when confirmed labels are expensive, class frequencies are highly asymmetric, and acquisition conditions vary in practice, while defining the scope of the conclusions supported by the current experiments.

### Related Works

Bolt-looseness diagnosis has been investigated using vibration response, percussion acoustics, acoustic emission, electromechanical impedance, guided waves, optical sensing, and magnetic measurements. Conventional systems generally follow a sensing–feature–classification pipeline: a structural response is acquired under an active or ambient excitation, diagnostic descriptors are extracted in the time, frequency, or time–frequency domain, and a statistical or machine-learning model maps those descriptors to a connection state. These methods remain attractive because their features can often be related to stiffness loss, damping variation, contact nonlinearity, or changes in the propagation path [1].

For vibration- and percussion-based monitoring, autocorrelation analysis [2] and wavelet-based feature extraction [3] have been used to enhance state-dependent components before classification. Acoustic-emission strategies have also been developed for multi-bolt structures [4,5]. Other studies have employed active piezoelectric sensing [6,7], distributed optical-fiber measurements [8], Lamb-wave phase changes [9], and magnetic measurements [10]. These approaches provide useful physical observability, but they frequently depend on dedicated sensing layouts, carefully designed excitation procedures, or handcrafted transformations whose parameters must be adjusted to a particular structure and operating condition.

Research specifically addressing transmission towers has analyzed state-dependent vibration features and fault localization under time-varying responses [11,21]. MFCC-based voiceprint representations [22] and acoustic-signature classifiers [23] have further demonstrated that impact responses contain discriminative information for tower-bolt assessment. Percussion monitoring under noise interference and limited samples has also been considered [24]. Collectively, these studies support the feasibility of non-destructive bolt diagnosis, but their performance is still affected by the tower topology, impact location, sensor location, propagation distance, looseness pattern, and environmental noise.

This dependence is particularly important for field-scale transmission towers. A feature that is stable on a laboratory joint or a single tower configuration may change when the propagation path or local connection geometry changes. Signal decomposition and feature selection can suppress part of the nuisance variation, but they do not by themselves guarantee that the resulting decision boundary remains valid across heterogeneous acquisition conditions. This limitation has motivated the use of learned representations that can integrate local impact patterns with longer-range temporal dependencies.

Deep learning reduces the dependence on manually selected descriptors by learning hierarchical representations from waveforms, spectra, or time–frequency maps. Percussion-based convolutional and recurrent models have been investigated for early and multi-bolt looseness detection [17,18], while graph convolution has been introduced to exploit the relationships among multiple sensing points [25]. Convolutional frameworks have also been applied to prefabricated spatial structures [19], and reconstruction or multitask learning has been combined with impedance measurements for temperature compensation and automated assessment [26,27].

Recent studies have further incorporated attention, graph reasoning, and reconstruction objectives. Representative examples include attention-enhanced dense convolution for percussion signals [28], multivariate active-sensing models for looseness localization [29], attention-based sequence modeling for adaptive anomaly recognition [30], and spectrogram-image classification of bolt and nut states [31]. Explainable artificial intelligence has been used to examine damage decisions [32], and transfer learning has been explored to improve generalization across acoustic-emission conditions [33]. These advances show that learned representations can improve diagnostic capacity, but most methods still rely on fully labeled training data collected under a limited set of structural and acquisition configurations.

Our previous supervised study learned multi-pattern vibration representations for bolt-looseness detection across multiple in-service transmission towers [20]. That study established the value of heterogeneous tower data, but it also exposed a practical labeling bottleneck. Loose configurations can be generated in many combinations on controllable test towers, whereas a fully tightened tower provides only a restricted set of tight-state patterns. Deliberately changing bolt states on operating towers is costly and potentially hazardous. Consequently, the labeled data are not only expensive but also asymmetric in class frequency and acquisition coverage, while substantially more field responses can be collected without reliable state labels.

Hybrid model-based diagnosis provides a complementary perspective. Pérez-Pérez et al. [34] identify neuro-fuzzy qLPV wind-turbine models and use zonotopic observers to construct residual fault signatures under bounded disturbances. The present method instead learns a binary tower-bolt classifier from MFCCs and uses reconstruction only as auxiliary representation supervision; it does not identify a plant model, implement an observer bank, or provide a bounded-error isolation guarantee.

Self-supervised learning has recently shown that unlabeled signals can support representation learning in other machinery-diagnosis tasks. Time–frequency contrastive and predictive objectives have been used for vehicle and train bearing diagnosis [35,36], while compositional generalization [37] and data-driven sensor configuration [38,39,40] address related distribution and observability problems. Nevertheless, these methods were developed for different machines and fault mechanisms; they do not establish how unlabeled MFCC vibration segments should be used for bolt diagnosis under severe class imbalance and heterogeneous tower conditions.

The resulting gap is therefore not simply the absence of a deeper classifier. It is the need to exploit abundant unlabeled measurements without assigning potentially unreliable pseudo-labels, while preserving the diagnostic information contained in the limited labeled data. The present study addresses this gap by sharing a conformer encoder between supervised state classification and denoising/masked-reconstruction objectives. Labeled samples define the tight/loose decision boundary, whereas both labeled and unlabeled samples constrain the encoder through feature reconstruction. The experiments then isolate the contribution of the unlabeled branch and characterize performance across the observed acquisition conditions. This scope evaluates robustness within the available multi-condition dataset; it does not, by itself, establish generalization to an entirely unseen tower type.

## 2. Methods

### 2.1. Dataset Construction and Input Representation

The vibration data used in this study were collected from in-service steel lattice transmission towers using a standardized hammer-excitation protocol. The dataset combines two complementary acquisition regimes: controlled experiments with confirmed bolt-state labels and routine field measurements for which the bolt state was not reliably verified. Both regimes used the same basic sensing and excitation configuration so that labeled and unlabeled measurements retained comparable vibration characteristics. Representative transmission-tower structures and field environments are shown in Figure 2. The relative locations of the excitation point, potential loosened-bolt regions, and vibration sensor are schematically illustrated in Figure 3, while representative field acquisition photographs are presented in Figure 4.

An STMicroelectronics IIS3DWB tri-axial digital MEMS vibration sensor (STMicroelectronics, Geneva, Switzerland) operated at the ±16 g full-scale setting (0.488 mg/LSB sensitivity, fixed 26.667 kHz output data rate, and approximately 6 kHz nominal −3 dB signal bandwidth) and was rigidly attached to the lower part of a tower leg using a magnetic mounting base. The sensor was installed near a bolted connection at a position that remained accessible for field operation while providing sensitivity to vibration changes associated with the local connection condition. A handheld impact hammer equipped with a custom-machined convex steel tip was used to excite a nearby angle-steel member. The impact region was maintained within approximately 1.2 m of the accelerometer position to reduce variation in the excitation–sensor geometry across measurements. This arrangement follows the field protocol established in our previous work, in which hammer excitation and tri-axial acceleration sensing were used to acquire transient responses from in-service transmission towers [20].

As shown in Figure 5, the IIS3DWB provided synchronous tri-axial digital acceleration samples at its fixed output data rate of 26.667 kHz, referred to as approximately 27 kHz throughout this study. The impact force itself was not instrumented. Instead, repeatability was promoted by maintaining a consistent hammer configuration and impact procedure, together with operator training and post-acquisition signal-quality checks. The acquired waveforms were retained without amplitude normalization or additional digital filtering before feature extraction, thereby preserving the original measurements for subsequent standardized preprocessing. The principal instrumentation parameters are summarized in Table 1. The detailed data acquisition procedure is provided in the Supplementary Materials.

One triaxial sensor supplies the three synchronous acceleration channels required by the implemented model. This is the minimum configuration needed to reproduce the model input under the studied arrangement, rather than evidence that one sensor provides complete diagnostic coverage of every joint on an arbitrary tower. Reproduction of the acquisition arrangement requires rigid sensor mounting, confirmation of channel continuity, inspection for saturation or incomplete records, and documentation of the sensor position and excitation geometry.

The impact force was not measured using a force transducer. Consequently, the archived experiment does not establish a validated minimum or maximum hammer force, a force-repeatability tolerance, or a force-response transfer function. The reported calibrated torque wrench and nominal 15 N·m criterion were used to verify the binary experimental state and should not be interpreted as a universal service preload or as calibration of the accelerometer.

The field measurements were not acoustically or mechanically isolated, and the available archive does not provide a validated environmental-noise acceptance threshold. Background activity and excitation geometry should therefore be documented, while saturated, incomplete, or visibly corrupted recordings should be rejected. The numerical waveform-noise tests characterize controlled perturbations and a recorded-background proxy; they do not constitute certification under wind, rain, traffic, or construction noise.

For the labeled experimental data, bolt condition was deliberately established and verified before vibration acquisition. A fully tightened condition was first established as the baseline configuration. Loose configurations were subsequently generated by deliberately loosening selected bolts at different structural locations and in different numbers under the supervision of field personnel. Bolt condition was verified using a calibrated torque wrench according to the operational inspection criterion adopted by the collaborating maintenance team, with a nominal threshold of 15 N·m used to distinguish loose and tight conditions. Each vibration record was ultimately assigned the binary state attribute tight or loose.

In addition to the binary state label, condition metadata were retained for subsequent stratified evaluation. The field distance_level records propagation-distance categories A–F associated with the structural path between the excitation point and the loosened region. Because exact distance values are unavailable for a subset of historical records, the subsequent analysis uses the recorded A–F level rather than treating every group as an exact continuous-distance interval. The field num_loose_screws records the number of loosened bolts associated with each loose-state measurement. Neither variable is used as a prediction target during training; they are retained only for post hoc analysis of detection performance under different acquisition and looseness conditions.

The unlabeled measurements were acquired using the same basic vibration-sensing protocol but without deliberate bolt-state intervention or reliable state confirmation. These records therefore retain acquisition metadata but are assigned no_label rather than a tight or loose target. They provide additional observations of structural and acquisition variability and are used exclusively for self-supervised reconstruction during model training.

A common audit retains 70,409 labeled and 154,394 deduplicated unlabeled MFCC segments. Nineteen labeled identities with non-finite, unmatched, or uncertain waveform–feature correspondence are excluded. Exact normalized-feature duplicates are removed from the unlabeled pool, and no retained unlabeled feature is identical to a labeled feature.

The retained labeled archive spans five recorded tower-type categories: 25,807 segments for 500 kV type 1, 18,246 for 500 kV type 2, 15,832 for 500 kV type 3, 9644 for 220 kV type 4, and 880 for 220 kV type 5. These are metadata categories rather than certified counts of independent physical towers; type 5 contains only tight samples.

Each labeled sample contains a confirmed state attribute,

$$y_{i}=\left\{\begin{array}{c} 0,\;\mathrm{tight},\\ 1,\;\mathrm{loose}. \end{array}\right.$$

For the revised analysis, source records are joined with inferred minute-level acquisition identities and exact normalized-MFCC hashes to form conservative groups. Stratified group folds assign 42,421 segments to training, 13,934 to validation, and 14,054 to testing. Source records, inferred acquisition groups, and exact-feature identifiers do not cross these roles.

The split is retrospective because it was formed from the existing archive, but all revised configurations use the same frozen partition. The archive cannot recover undocumented physical-tower identities, so physical-tower independence cannot be certified even though the identifiable groups are disjoint.

The validation set alone is used for checkpoint selection, temperature scaling, and operating-threshold selection. The test set is used once per frozen model for final reporting and is not used for stopping, calibration, threshold changes, or hyperparameter changes. Because the partition was constructed from an archive used during earlier method development, it is not described as a prospective deployment test.

As shown in Figure 6, the revised training partition remains imbalanced, with 33,215 loose and 9206 tight segments. Equal-probability class sampling with replacement is therefore used during training; evaluation retains the natural composition of each frozen validation or test partition.

**Figure 6:**
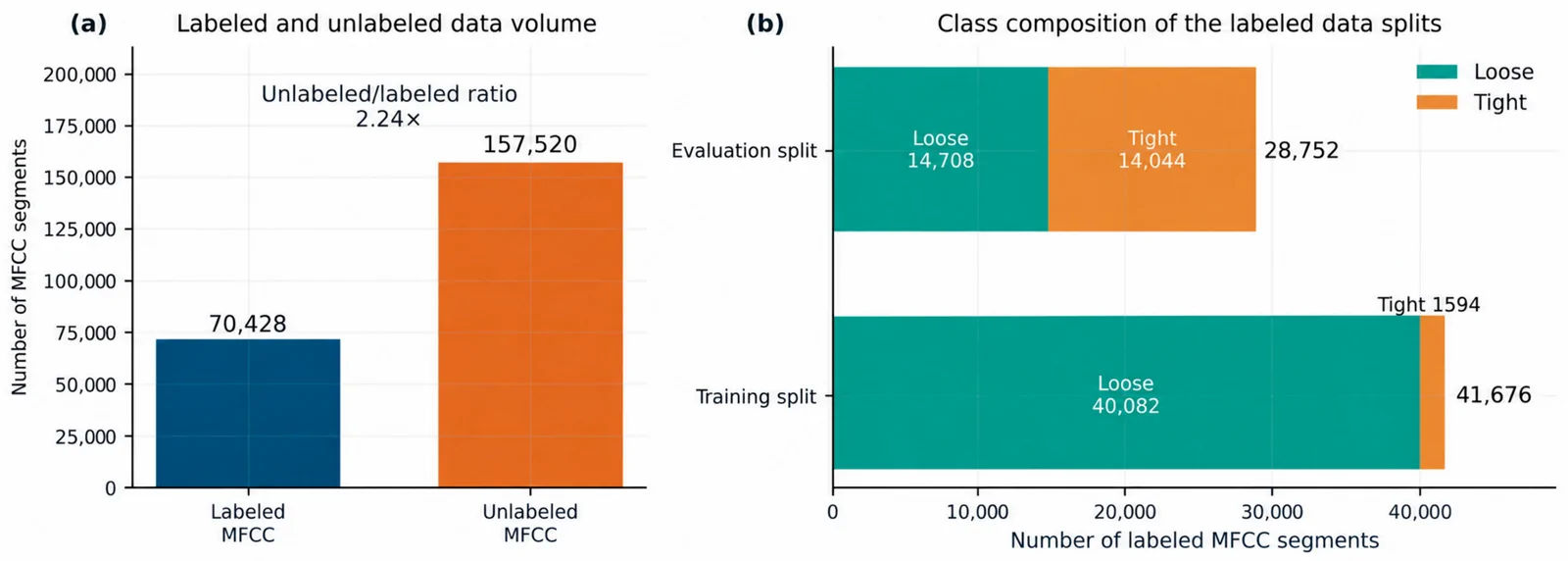
Original archive composition before the revised reproducibility audit. The unchanged diagram documents the initial two-role dataset description; the deduplicated training, validation, test, and unlabeled counts used for the revised analyses are reported in Table 2.

**Table 2:** Revised labeled partitions and deduplicated unlabeled pool. Groups are inferred acquisition units rather than certified physical towers.

Partition	Segments	Loose	Tight	Groups	Source Records
Training	42,421	33,215	9206	1332	3861
Validation	13,934	11,156	2778	444	1269
Test	14,054	10,403	3651	446	1281
Unlabeled	154,394	Unknown	Unknown	Not used for labeled splitting	Separate source pool

The audited unlabeled pool contains 154,394 distinct samples after exact normalized-feature deduplication. These samples retain no verified tight/loose target and are used only for denoising and masked-reconstruction objectives.

Because the physical bolt states of the unlabeled records were not reliably confirmed, their state attribute is set to no_label and they are never assigned a tight or loose pseudo-label. The unlabeled data are not divided into classification training and test subsets and do not contribute to classification metrics. They enter training only through the denoising and masked-reconstruction objectives described in Section 4.

Additional metadata are retained for the loose-state samples to support condition-stratified evaluation. The recorded distance_level field defines propagation-distance groups A–F. Independently, the num_loose_screws field records the number of loosened bolts. For the latter analysis, loose evaluation samples are grouped into 1–4, 5–9, 10–14, 15–19, and ≥20 loosened bolts. These fields are used only to stratify already confirmed loose samples after inference. The network remains a binary tight/loose classifier and does not predict propagation distance or the number of loosened bolts.

The model does not operate directly on the high-sampling-rate acceleration waveform. Instead, each extracted tri-axial vibration segment is transformed into a fixed-size MFCC representation that retains short-time spectral-envelope information while substantially reducing the dimensionality of the input.

For offline preprocessing, the implementation detects positive peaks on the Z channel at 0.6 times the smallest of the three channel maxima, enforces a two-second minimum separation, and retains up to ten peaks. Explicit first, interior, and last boundary rules produce a verified 69,632-sample waveform per axis.

MFCCs are computed independently for each axis with librosa 0.11.0 using a centered 16,384-sample Hann STFT, hop length 4096, constant zero padding, power exponent 2, 128 Slaney-normalized non-HTK Mel bands from 0 to fs/2, dB [1] with amin 10^−10^ and an 80 dB floor, and an orthonormal type-II DCT. Coefficients 0–12 are retained.

The centered transform produces 18 frames. The implementation retains zero-based frames 0–13, then computes first- and second-order terms with nine-frame Savitzky–Golay differentiation and interpolation. Concatenating 13 static, 13 delta, and 13 delta–delta coefficients for each axis gives three 14 × 39 blocks and the final 42 × 39 array. This choice reproduces the archived feature implementation; it was not selected through a search on the revised test set.

(1) $$M^{\left(a\right)}=\left[C^{\left(a\right)},\Delta C^{\left(a\right)},\Delta^{2C^{\left(a\right)}}\right]\in R^{14\times 39},\;\;\;\;\;a\in \{X,Y,Z\}$$

where ***C***_(*a*)_∈ℝ^14×13^ denotes the static MFCC coefficients, while Δ***C***(*a*) and Δ^2^***C***(*a*) denote the corresponding first-and second-order dynamic terms.

The three axis-specific matrices are concatenated along the first dimension,

***X*** = *Concat*(***M^X^***|, |***M^Y^***|, |***M^Z^***) ∈ ℝ^42×39^ (2)

The resulting array has a clear block structure: rows 1–14 correspond to the *X*-axis frames, rows 15–28 to the *Y*-axis frames, and rows 29–42 to the *Z*-axis frames, while the three 13-dimensional column blocks contain the static, delta, and delta–delta coefficients, respectively. The 42-position dimension should therefore be interpreted as an ordered sequence of axis-specific temporal frames, rather than as 42 consecutive temporal frames from a single vibration channel. Temporal adjacency exists within each 14-frame axis block, whereas transitions between adjacent axis blocks do not represent continuous physical time.

Each ℝ^42×39^ array ***i***s subsequently standardized independently. For sample i,

(3) $$\bar{X_{i}}=\frac{X_{i}-\mu_{i}}{\sigma_{i}}$$

where *μ**_i_*** and *σ**_i_*** are the mean and standard deviation calculated over all 1638 elements of the sample. Non-finite values are replaced safely in the implementation, and normalization is applied only when the standard deviation exceeds the numerical-stability threshold of 10^−6^. This per-sample transformation places MFCC inputs on a comparable numerical scale and emphasizes relative within-sample structure. It also suppresses absolute vibration-energy differences; consequently, the network output should not be interpreted as a direct estimate of vibration amplitude, bolt preload, or connection stiffness.

Labeled and unlabeled MFCCs have different but complementary roles during training. A labeled sample provides both an input ${\bar{X}}_{i}$ and the binary state target ***y_i_***, and therefore contributes to the supervised tight/loose classification objective as well as the auxiliary reconstruction objectives. An unlabeled sample provides only its MFCC input and is assigned the internal state value no_label; it does not contribute to any classification cross-entropy term. Instead, the original MFCC array itself provides the target for artificial denoising and masked-reconstruction tasks.

Both labeled and unlabeled samples are processed by the shared conformer encoder. Classification losses are computed only for labeled data, whereas denoising and masked-reconstruction losses are evaluated for both data pools. No state pseudo-label is generated for an unlabeled sample. In this way, the 154,394 unlabeled field measurements provide structural supervision for representation learning without introducing an unverified tight or loose class assignment.

### 2.2. Proposed Method

We propose a semi-supervised diagnostic architecture for MFCC vibration features, referred to as the conformer-denoising MFCC network. The method addresses three characteristics of the data regime: severe class imbalance in the labeled training split, incomplete coverage of acquisition conditions by confirmed labels, and the availability of a substantially larger unlabeled pool. Rather than generating tight or loose pseudo-labels for unlabeled segments, the method integrates state classification and MFCC reconstruction through one shared encoder. Labeled data supervise the diagnostic boundary, while both labeled and unlabeled data supervise recovery over the implemented token-position and feature-coordinate axes.

The network comprises an MFCC token-array embedding, a conformer encoder, a token-level denoising head, a masked-reconstruction head, and a state classifier. The embedding maps each of the 42 axis-specific temporal token positions into a common latent space. The conformer encoder combines attention across token positions with convolution over local adjacency within the axis-specific token sequence. The denoising branch reconstructs the corresponding clean pre-corruption array from a Gaussian-corrupted view, whereas the masked branch recovers contiguous missing regions along the token-position and feature-coordinate axes. The classifier predicts the tight or loose state from pooled encoder features. Because all objectives share the main encoder, reconstruction supervision derived from unlabeled samples directly updates the feature extractor used by the diagnostic task. The complete training and inference pathways are shown in Figure 7.

The token design follows the stored feature layout: each of the 42 tokens is one temporal frame within one of three 14-frame axis blocks, and its 39 coordinates contain static, delta, and delta–delta coefficients. This preserves within-axis local order while allowing attention across all positions; axis boundaries are not interpreted as continuous physical time. Architectural dimensions and training settings are listed in Table 3 and Table 4.

Let the standardized MFCC input be $\bar{x}\in R^{T\times F}$, where *T* = 42 indexes the axis-specific temporal token positions and *F* = 39 indexes the MFCC feature coordinates. A linear layer first maps each token-position vector to a latent token, after which a learnable positional embedding is added:

(4) $$H^{\left(0\right)}=\mathrm{Embed}\left(\bar{x}\right)+P$$

where H^(0)^ is the initial token array and P preserves the implemented axis-specific temporal token ordering. Temporal adjacency is preserved within each 14-frame axis block, whereas the boundaries between adjacent axis blocks correspond to changes in acceleration axis rather than continuous physical time.

The tokens then pass through a stack of conformer blocks. The k-th block is expressed as

(5) $$\begin{array}{l} {\tilde{H}}^{\left(k\right)}=H^{\left(k-1\right)}+\frac{1}{2}\cdot {\mathrm{FFN}}_{1}\left(\mathrm{LN}\left(H^{\left(k-1\right)}\right)\right)\\ {\hat{H}}^{\left(k\right)}={\tilde{H}}^{\left(k\right)}+\mathrm{MHA}\left(\mathrm{LN}\left({\tilde{H}}^{\left(k\right)}\right)\right)\\ {\bar{H}}^{\left(k\right)}={\hat{H}}^{\left(k\right)}+\mathrm{Conv}\left(\mathrm{LN}\left({\hat{H}}^{\left(k\right)}\right)\right)\\ H^{\left(k\right)}=\mathrm{LN}\left[{\bar{H}}^{\left(k\right)}+\frac{1}{2}\cdot {\mathrm{FFN}}_{2}\left(\mathrm{LN}\left({\bar{H}}^{\left(k\right)}\right)\right)\right] \end{array}$$

Here, FFN, MHA, Conv, and LN denote the feed-forward, multi-head self-attention, convolutional, and layer-normalization modules, respectively. Half-step residual scaling distributes the feed-forward transformation around the attention and convolutional operations. Self-attention models dependencies across non-adjacent token positions, whereas the convolutional module, which contains pointwise convolution, gating, depthwise convolution, batch normalization, and nonlinear mapping, models local adjacency within the implemented token sequence. Their combination learns global and local relationships in the implemented array. These relationships are useful computational features, but they are not interpreted here as strictly continuous temporal evolution or physical decay.

After the final conformer block, mean pooling over token positions produces a segment-level representation:

(6) $$h=\mathrm{Pool}\left(H\right)=\frac{1}{T}\sum_{t=1}^{T}H_{t}$$

The pooled vector is used for state classification. The unpooled representation retains alignment with the 42 input token positions and is used by the token-level reconstruction heads. In Equation (6), the subscript t is a token-position index rather than a claim of verified continuous time ordering.

To exploit structural information in both labeled and unlabeled MFCCs, a Gaussian-corruption denoising task is constructed. For labeled training, MixUp is applied before both classification and reconstruction. Let *x_mix_* = *λx_a_* + (1 − *λ*)*x_b_*, where *λ* is sampled from the MixUp distribution, and let the corresponding soft classification target interpolate *y_a_* and *y_b_* using the same coefficient. In the following formulation, *x* = *x_mix_* for labeled training, whereas *x* = *u* is the original unlabeled MFCC for unlabeled reconstruction. A corruption coefficient *t* is then sampled and a perturbed input is formed as

**z** = *t***x** + (1 − *t*)***ε***,     ***ε***∼𝒩(0,**I**) (7)

where *t*∈(0,1] controls the relative contributions of the clean pre-corruption input and random noise. Values close to one preserve most of that input, whereas smaller values produce stronger corruption.

The corrupted input is first processed by the shared conformer encoder to obtain a token array *H_z_* = *E_θ_*_(*z*)_. Embeddings of the noise level *t* and condition *c* are then added to these encoded tokens immediately before the token-level residual head. They do not enter the conformer blocks that produce the corrupted-input representation *h_n_*. For labeled MixUp samples, *c* follows the primary label *y_a_* and is independently replaced by the null condition with probability 0.1, whereas the classification loss uses the interpolated targets *y_a_* and *y_b_*. Unlabeled samples always use the null condition. The denoising head predicts a 39-dimensional residual at each token position:

(8) $$\hat{x}=z+\left(1-t\right)\Delta_{\theta }\left(z,t,c\right)$$

where Δ*_θ_* is the token-level residual predictor. Token-aligned recovery preserves one-to-one correspondence between positions in the input token–feature array and the reconstructed output, in contrast to reconstructing the complete MFCC from a single compressed global vector.

The factor 1 − *t* scales the permitted correction. When the input is nearly clean, only a small residual is applied; under stronger corruption, a larger correction is allowed. The denoising loss is the mean squared error between the reconstruction and the clean pre-corruption target:

(9) $$L_{\mathrm{den}}=\mathrm{MSE}\left(\hat{x},x\right)$$

Because the clean pre-corruption input defines the target—*x_mix_* for labeled training and the original MFCC for unlabeled training—the objective can be evaluated for both data pools.

Gaussian denoising addresses continuous-valued perturbations. To additionally train recovery from missing coordinates, contiguous regions are randomly masked along the token-position and feature-coordinate axes of the implemented array, producing *m*(*x*). Here, as in the denoising branch, *x* denotes *x_mix_* for labeled training and the original MFCC for unlabeled training. The model predicts the complete pre-mask input from the visible array context:

(10) $$\tilde{x}=R_{\theta }\left(m\left(x\right)\right)$$

where *R_θ_* comprises the shared conformer encoder and token-level reconstruction head. The corresponding objective is

(11) $$L_{\mathrm{mask}}=\mathrm{MSE}\left(\tilde{x},x\right)$$

Contiguous masking prevents the encoder from relying exclusively on a small set of fixed token positions or feature coordinates and requires the missing values to be inferred from the remaining array context. Denoising and masking thus provide complementary supervision for continuous-valued corruption and coordinate removal. Both objectives are applied to labeled and unlabeled samples. The masks are not interpreted as verified continuous time intervals or physical frequency bands.

Artificial corruption supports reconstruction learning and input augmentation, but routine diagnosis uses MFCCs without added Gaussian noise. The model therefore includes both a corrupted-input classification pathway and an uncorrupted-input classification pathway. Here, uncorrupted denotes the absence of the Gaussian mixture in Equation (7); SpecAugment can still be applied to this pathway during training, as specified in Section 3.1.

For the corrupted pathway, segment-level representations are obtained from the corrupted input *z* and its reconstruction $\hat{x}$:

(12) $$h_{n}=\mathrm{Pool}\left(E_{\theta }\left(z\right)\right),\;\;\;\;\;h_{d}=\mathrm{Pool}\left(E_{\theta }\left(\hat{x}\right)\right)$$

The two representations are concatenated and passed to the state classifier:

(13) $$\hat{y}=C_{\theta }\left(\left[h_{n};h_{d}\right]\right)$$

The corrupted representation retains discriminative information present in the observation, while the reconstructed representation provides features after structural recovery. Their joint use links classification and denoising within the shared encoder during training.

A second labeled pathway processes the uncorrupted input, optionally after clean-view masking. At clean inference, t = 1 makes the residual multiplier zero, so the reconstructed input equals the original MFCC and both pooled representations equal h. The implemented shortcut therefore evaluates the shared encoder once and supplies the unchanged [h, h] vector to the original 320-dimensional LayerNorm and classifier. It removes duplicated computation without changing the trained feature structure.

The two labeled classification pathways yield the corrupted-input cross-entropy loss ℒ*_ce_* and the uncorrupted-input classification loss ℒ*_clean_*.

The network is trained by jointly optimizing state classification, denoising, and masked reconstruction. Superscripts l and u denote labeled and unlabeled data, respectively:

(14) $$\begin{array}{cc} L= & \lambda_{\mathrm{ce}}L_{\mathrm{ce}}+\lambda_{\mathrm{clean}}L_{\mathrm{clean}}\\ & +\lambda_{\mathrm{den}}L_{\mathrm{den}}^{l}+\lambda_{\mathrm{uden}}L_{\mathrm{den}}^{u}\\ & +\lambda_{\mathrm{mask}}L_{\mathrm{mask}}^{l}+\lambda_{\mathrm{umask}}L_{\mathrm{mask}}^{u} \end{array}$$

The classification terms are computed only for labeled samples. The remaining four terms correspond to denoising and masked reconstruction over the labeled and unlabeled pools. This objective assigns distinct but complementary roles to the data: confirmed labels define the diagnostic boundary, while all available MFCCs constrain the representation of recoverable signal structure. Loss weights and implementation details are given in Section 3.1.

## 3. Experimental Results

### 3.1. Experimental Setup and Implementation Details

The model input has a fixed size of 42 × 39, and the token embedding dimension is 160. The conformer encoder contains three blocks with four attention heads per block, a depthwise-convolution kernel size of 7, and a dropout rate of 0.15. The current implementation instantiates 2,079,584 parameters and produces a checkpoint of approximately 7.98 MiB. Approximately 2,017,920 parameters participate in the classification, denoising, and masked-reconstruction objectives. A further 61,664 parameters belong to an auxiliary projection branch that is not included in the objectives reported in this study; consequently, that branch is excluded from the representation analysis.

Neural models use AdamW with learning rate 3 × 10^−4^, weight decay 10^−4^, cosine scheduling, gradient clipping at norm 1.0, and automatic mixed precision. Complete-data runs use 30 epochs and reduced-label runs use 80; labeled and unlabeled batch sizes are 512. Complete-data and label-budget comparisons use seeds 17, 29, 43, 71, and 101, while held-out-type tests use seeds 17, 29, and 43.

Class-balanced weighted sampling is applied to the labeled training split, without an additional class-weighted loss. The supervised pathways use MixUp with *α* = 0.2 and label smoothing of 0.03. MixUp precedes Gaussian corruption, masked reconstruction, and both labeled classification pathways; consequently, the mixed MFCC is also the target of the labeled denoising and masked-reconstruction losses. For Gaussian corruption, *r*∼*N*(−0.8,0.8^2^) is sampled independently and *t* = *clamp*(*sigmoid*(*r*),0.05,1.0); the Gaussian noise scale is 1.0. The implementation routine named SpecAugment is applied to the uncorrupted-input classification pathway with probability 0.2, using one token-axis mask and one feature-axis mask with independently sampled widths from 1 to 4 and no additional magnitude noise. Each masked-reconstruction input uses two token-axis masks and two feature-axis masks with widths from 1 to 8, followed by additive perturbation with standard deviation 0.05. The primary labeled condition is replaced by the null condition with probability 0.1, whereas unlabeled samples always use the null condition. The final model does not use pseudo-labels for unlabeled data.

The layered architecture is summarized in Table 3. The principal optimization, augmentation, reconstruction, loss-weight, and selection settings are consolidated in Table 4.

After each epoch, the earliest maximum validation macro-F1 checkpoint is retained using threshold 0.5. Temperature scaling minimizes validation negative log likelihood over T ∈ [0.1, 10], after which the operating threshold is selected on validation from 0.05 to 0.95 in increments of 0.05, using the smallest threshold in a tie. The fitted temperature and threshold are frozen before test or later-cohort inference.

All evaluations treat looseness as the positive class. In addition to accuracy, balanced accuracy, class-wise precision and recall, macro-F1, ROC–AUC, and average precision for both classes, the revised analysis reports specificity, false-positive rate, Brier score, negative log likelihood, and 15-bin equal-width expected calibration error. Means and standard deviations summarize training-seed variability; paired 95% t intervals, paired tests, exact sign-flip probabilities, and paired standardized differences quantify matched comparisons.

Experiments were conducted on Ubuntu 24.04.4 LTS using Python 3.11.15, PyTorch 2.6.0+cu124, CUDA 12.4, cuDNN 9.1.0, NumPy 2.4.4, and scikit-learn 1.8.0. The workstation contains an Intel Core i9-14900KF processor and two NVIDIA GeForce RTX 3090 GPUs with 24 GB of memory each. A single GPU was used for each training or evaluation run.

### 3.2. Comparison with Representative Benchmark Methods

The revised complete-data comparison uses the same grouped partition, epoch schedule, validation selection, and test protocol for every neural configuration. It includes the full model, labeled reconstruction, clean classification, clean/noisy classification without explicit reconstruction, separate unlabeled denoising and masking additions, removal of each principal augmentation, a matched transformer, a single-representation classifier, a legacy-layout control, and documented CNN, LSTM, VMD, SVC, and XGBoost baselines.

For every model, checkpoint selection, temperature fitting, and operating-threshold selection use validation data only. Test and later-cohort outputs are produced with the frozen validation choices. Five-seed results are reported where stochastic training is used; deterministic models retain zero seed variability.

Published baselines are adapted only where required by the common input and evaluation interface. Their scores represent documented implementations on the present audited cohorts rather than exact reproductions of results from different structures and acquisition conditions.

As, shown in Figure 8, the full model obtains test macro-F1 0.9124 ± 0.0033 and ROC–AUC 0.9692 ± 0.0025. The labeled-reconstruction control obtains 0.9032 ± 0.0135 and 0.9644 ± 0.0074. The unlabeled-masking-only variant has the highest mean macro-F1, 0.9144 ± 0.0064; therefore, combining both unlabeled objectives is not claimed to be optimal.

**Figure 8:**
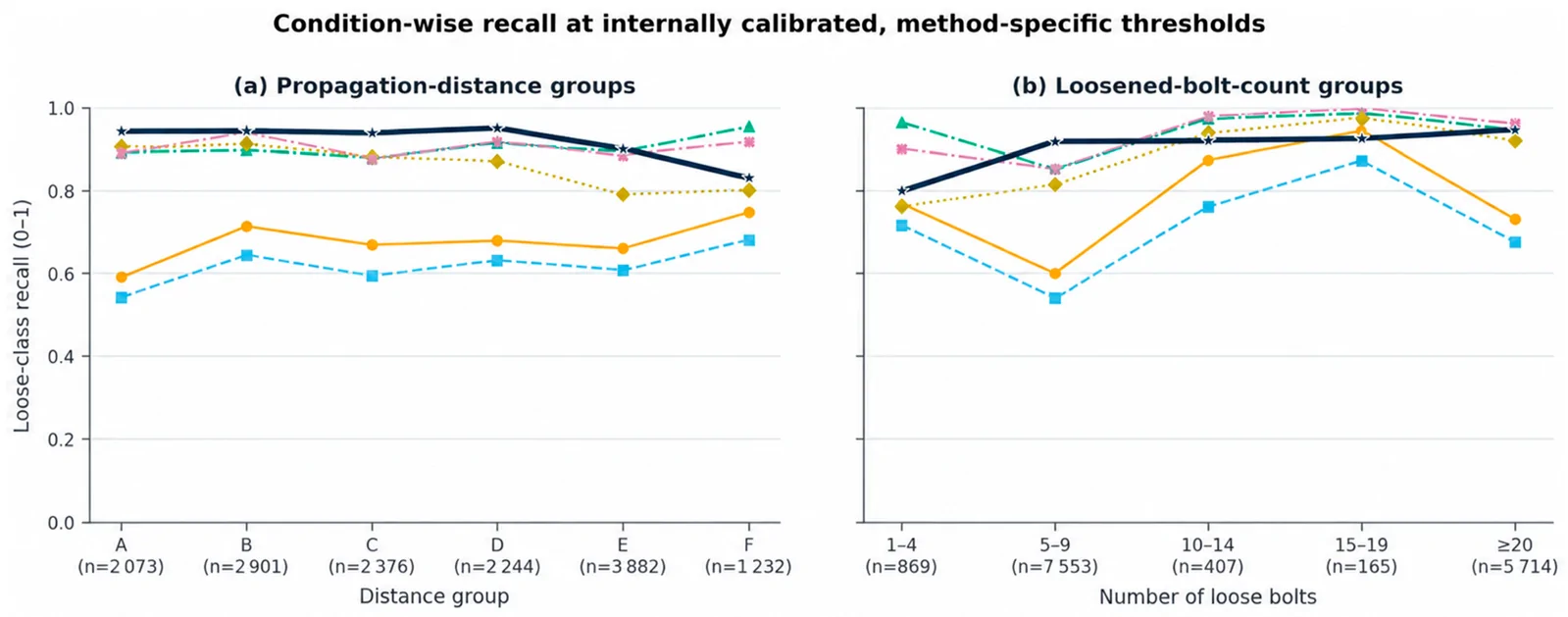
Original single-run condition-wise benchmark visualization retained without alteration. It documents the initial internally calibrated analysis; all revised comparative claims [26,27,28,29,30,31] use the grouped validation/test protocol and multi-seed results in Table 5, Table 6, Table 7 and Table 8.

**Table 5:** Controlled complete-data and benchmark results under fixed validation selection. Values are mean ± SD across five seeds where applicable.

Configuration	Test Macro-F1	Test ROC AUC	External Macro-F1	External ROC AUC
Full model	0.9124 ± 0.0033	0.9692 ± 0.0025	0.4674 ± 0.0171	0.4812 ± 0.0249
Labeled reconstruction	0.9032 ± 0.0135	0.9644 ± 0.0074	0.4517 ± 0.0299	0.4772 ± 0.0275
Clean classification only	0.9039 ± 0.0131	0.9655 ± 0.0075	0.4764 ± 0.0168	0.5020 ± 0.0147
Clean and noisy classification	0.9080 ± 0.0041	0.9677 ± 0.0022	0.4641 ± 0.0169	0.4851 ± 0.0237
Add unlabeled denoising	0.9104 ± 0.0090	0.9688 ± 0.0027	0.4700 ± 0.0201	0.4887 ± 0.0217
Add unlabeled masking	0.9144 ± 0.0064	0.9696 ± 0.0018	0.4638 ± 0.0353	0.4795 ± 0.0403
No MixUp	0.9097 ± 0.0063	0.9686 ± 0.0021	0.4715 ± 0.0297	0.4917 ± 0.0302
No clean-view masking	0.9096 ± 0.0074	0.9647 ± 0.0025	0.4584 ± 0.0345	0.4732 ± 0.0357
No label smoothing	0.9075 ± 0.0063	0.9685 ± 0.0032	0.4745 ± 0.0145	0.4890 ± 0.0112
Matched Transformer	0.8602 ± 0.0137	0.9444 ± 0.0117	0.4667 ± 0.0233	0.4810 ± 0.0306
Single representation	0.9098 ± 0.0087	0.9664 ± 0.0064	0.4436 ± 0.0156	0.4594 ± 0.0193
Legacy feature layout	0.8988 ± 0.0084	0.9639 ± 0.0031	0.4491 ± 0.0091	0.4542 ± 0.0176
Raw 1D CNN	0.8382 ± 0.0049	0.8933 ± 0.0041	0.4130 ± 0.0146	0.5636 ± 0.0308
MFCC LSTM	0.8885 ± 0.0089	0.9632 ± 0.0049	0.4983 ± 0.0373	0.5101 ± 0.0169
VMD MLP	0.7365 ± 0.0070	0.8212 ± 0.0020	0.5021 ± 0.0107	0.5185 ± 0.0108
VMD SVC	0.6067 ± 0.0000	0.6739 ± 0.0000	0.4294 ± 0.0000	0.4628 ± 0.0000
XGBoost	0.9000 ± 0.0020	0.9611 ± 0.0006	0.4338 ± 0.0104	0.4669 ± 0.0133

**Table 6:** Full-model diagnostic and calibration metrics. Calibration parameters and thresholds are fitted on validation only; values are mean ± SD across five seeds.

Metric	2025 Raw	2025 Calibrated	2026 Raw	2026 Calibrated
Macro-F1	0.8994 ± 0.0064	0.9124 ± 0.0033	0.4687 ± 0.0290	0.4674 ± 0.0171
Accuracy	0.9219 ± 0.0057	0.9365 ± 0.0020	0.4712 ± 0.0273	0.4803 ± 0.0144
Balanced accuracy	0.9032 ± 0.0032	0.8897 ± 0.0064	0.4712 ± 0.0273	0.4803 ± 0.0144
Loose precision	0.9519 ± 0.0019	0.9313 ± 0.0049	0.4752 ± 0.0230	0.4844 ± 0.0114
Loose recall	0.9420 ± 0.0092	0.9871 ± 0.0048	0.5350 ± 0.0168	0.6247 ± 0.0660
Tight specificity	0.8644 ± 0.0066	0.7924 ± 0.0169	0.4074 ± 0.0518	0.3359 ± 0.0613
False-positive rate	0.1356 ± 0.0066	0.2076 ± 0.0169	0.5926 ± 0.0518	0.6641 ± 0.0613
ROC AUC	0.9692 ± 0.0025	0.9692 ± 0.0025	0.4812 ± 0.0249	0.4812 ± 0.0249
Loose average precision	0.9869 ± 0.0018	0.9869 ± 0.0018	0.4863 ± 0.0283	0.4863 ± 0.0283
Tight average precision	0.9427 ± 0.0034	0.9427 ± 0.0034	0.5341 ± 0.0185	0.5341 ± 0.0185
Brier score	0.0578 ± 0.0036	0.0577 ± 0.0037	0.4210 ± 0.0136	0.4579 ± 0.0144
Negative log likelihood	0.1996 ± 0.0121	0.1920 ± 0.0102	1.2997 ± 0.0892	1.6569 ± 0.1613
ECE with 15 bins	0.0594 ± 0.0100	0.0414 ± 0.0047	0.4078 ± 0.0185	0.4504 ± 0.0180

**Table 7:** Paired five-seed comparison of the full model and labeled-reconstruction control.

Configuration	Macro-F1	ROC–AUC	Paired Difference	95% Seed Interval
Full model	0.9124 ± 0.0033	0.9692 ± 0.0025	+0.00915	[−0.00503, +0.02333]
Labeled reconstruction	0.9032 ± 0.0135	0.9644 ± 0.0074	Reference	—

**Table 8:** Label-budget results across five paired seeds. Intervals describe the paired mean difference across training seeds.

Labels Per Class	Full Macro-F1	Labeled Macro-F1	Paired Difference	95% Seed Interval
20	0.5792 ± 0.0107	0.5805 ± 0.0091	−0.0012	[−0.0143, +0.0119]
50	0.5861 ± 0.0163	0.5910 ± 0.0161	−0.0049	[−0.0132, +0.0034]
100	0.6033 ± 0.0300	0.6030 ± 0.0341	+0.0003	[−0.0065, +0.0070]
200	0.6239 ± 0.0166	0.6224 ± 0.0221	+0.0015	[−0.0203, +0.0233]
500	0.6879 ± 0.0168	0.6912 ± 0.0201	−0.0033	[−0.0121, +0.0055]
1594	0.7864 ± 0.0171	0.7917 ± 0.0167	−0.0053	[−0.0143, +0.0038]

The matched transformer reaches test macro-F1 0.8602 ± 0.0137 under the same protocol, while the single-representation classifier reaches 0.9098 ± 0.0087. The later-cohort results are low for all configurations, preventing a general cross-collection superiority claim.

Small differences among the conformer controls are interpreted with paired seed intervals rather than by rank alone. The controlled comparisons isolate objectives or architecture more directly than the earlier mixed-protocol development table.

### 3.3. Overall Diagnostic Performance and Threshold Sensitivity

Table 6 reports class-wise diagnostic and calibration results for the full model on the 14,054-segment grouped test and the later 2800-segment cohort. On the grouped test, validation calibration gives loose recall 0.9871 ± 0.0048, tight specificity 0.7924 ± 0.0169, false-positive rate 0.2076 ± 0.0169, macro-F1 0.9124 ± 0.0033, and ROC–AUC 0.9692 ± 0.0025.

As shown in Figure 9, the calibrated grouped-test Brier score, negative log likelihood, and ECE are 0.0577 ± 0.0037, 0.1920 ± 0.0102, and 0.0414 ± 0.0047, respectively. Calibration does not improve every metric or transfer setting, so raw and calibrated values are both reported.

As shown in Figure 10, the operating threshold controls the trade-off between loose-state detection and false alarms among tight samples. This is selected on validation and is frozen for final inference; any deployment threshold would additionally require target-population prevalence and cost information unavailable here.

### 3.4. Effect of Unlabeled Reconstruction and Label-Budget Analysis

The primary reconstruction comparison uses matched full and labeled-reconstruction models at seeds 17, 29, 43, 71, and 101. Within each pair, the partition, labeled subset, data streams, evaluation frequency, validation selection, and test samples are identical.

The paired macro-F1 difference is +0.00915 with seed SD 0.01142 and a 95% interval of [−0.00503, +0.02333]. The paired *t*-test gives *p* = 0.1477, the exact sign-flip test gives *p* = 0.25, and d_z = 0.801; three of five pairs favor the full model. The observed mean improvement is therefore not statistically conclusive across these five seeds.

Reduced-label comparisons use 20, 50, 100, 200, 500, and 1594 labeled samples per class, five fixed per-seed subset draws, batch size 512, 80 epochs, and a shared 30,000-sample deduplicated unlabeled pool. Each pair uses identical labeled samples and validation-only selection.

Every label-budget paired interval includes zero. Mean full-minus-labeled differences are negative at 20, 50, 500, and 1594 samples per class and near zero at 100 and 200. The revised results therefore do not support a general claim that unlabeled reconstruction reduces annotation requirements.

As shown in Figure 11, the largest balanced subset contains 3188 labeled segments, compared with 42,421 segments in the revised complete-data training partition. It also removes much of the loose-state and condition diversity, so the reduced-label experiment addresses a different regime from the complete-data comparison.

### 3.5. Held-Out Tower-Type and Temporal Transfer

Because certified physical-tower identities are unavailable, physical-tower leave-one-out evaluation cannot be supported. We instead perform leave-one-recorded-tower-type-out stress tests with three seeds, ensuring that the held-out category supplies the test samples while the remaining categories supply training and validation data.

As shown in Table 9, full-model macro-F1 ranges from 0.3309 to 0.4964 for the four held-out types containing both classes. Type 5 contains 880 tight samples only, so two-class macro-F1 and ROC–AUC are undefined. On the later 2800-segment cohort, the full model reaches macro-F1 0.4674 ± 0.0171. Neither analysis establishes unseen physical-tower performance.

### 3.6. Condition Subgroups and Waveform Stress Tests

Propagation-distance and loosened-bolt-count analyses are restricted to loose samples in the revised grouped test. They report recall for the full and labeled-reconstruction models across five seeds; no subgroup contains tight examples for estimating condition-specific specificity.

Recall remains high across the archived distance categories, but group size, tower type, excitation geometry, and acquisition membership differ. These observational categories cannot isolate a causal propagation-distance effect or define a validated sensor-spacing limit.

The same revised loose-test cohort is also grouped by the recorded number of loosened bolts. Recall remains high across the available count bins, but the binary score is not calibrated as an estimator of bolt count.

Differences among count bins are descriptive and potentially confounded with tower type, propagation path, and acquisition design. They do not support a causal effect of bolt count or a count-regression interpretation.

To evaluate physical-signal sensitivity, Gaussian noise and a recorded-background vibration proxy are added to aligned triaxial waveforms before MFCC extraction at joint SNR values of 20, 10, and 0 dB. Three fixed realization seeds are shared by all evaluated models; operating thresholds remain fixed from validation.

As shown in Figure 12 and Figure 13, the full-model macro-F1 falls to 0.4958 under 20 dB Gaussian waveform noise and to 0.5355 under the 20 dB recorded-background proxy, with further degradation at lower SNR. These controlled additions do not define field-noise acceptance limits and show that feature reconstruction alone does not establish waveform-level robustness.

**Figure 12:**
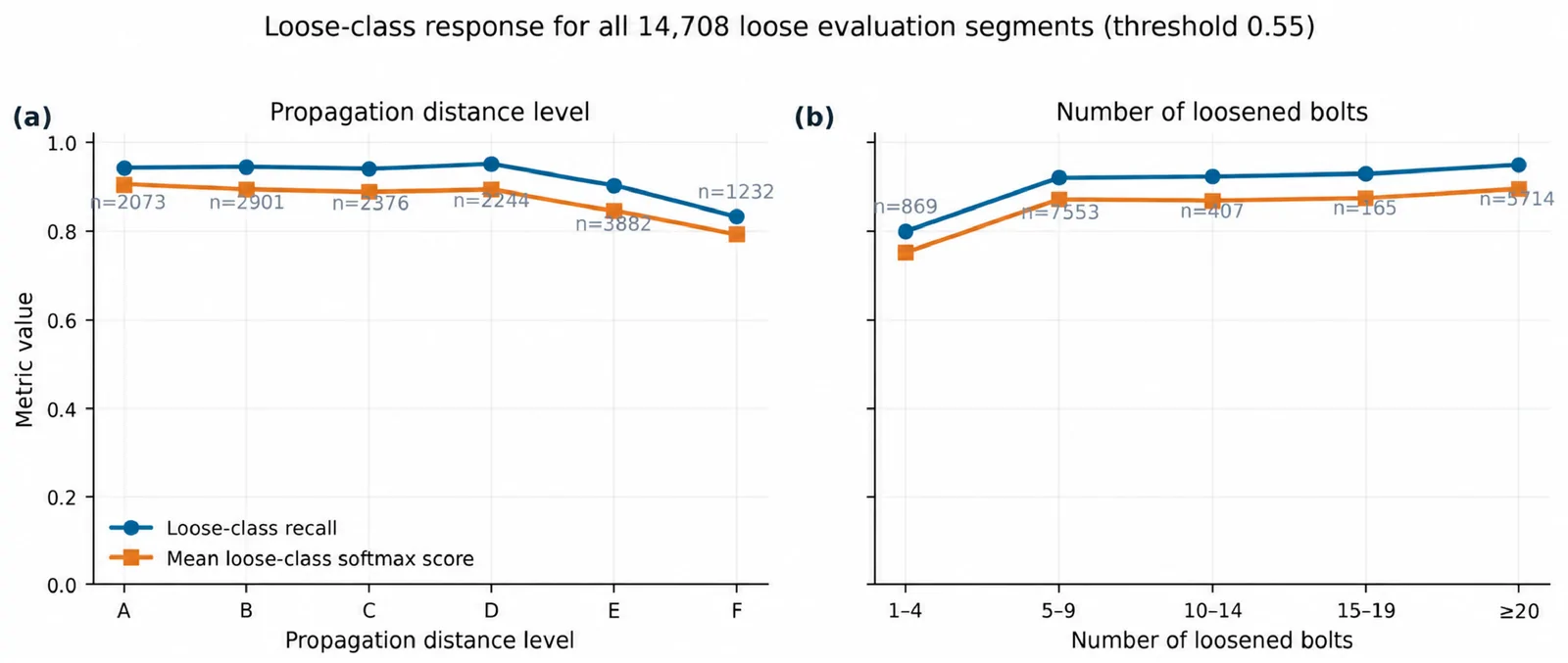
Original single-run subgroup visualization retained without alteration. Revised five-seed subgroup summaries are reported in Table 10 and Table 11 and are interpreted descriptively.

**Figure 13:**
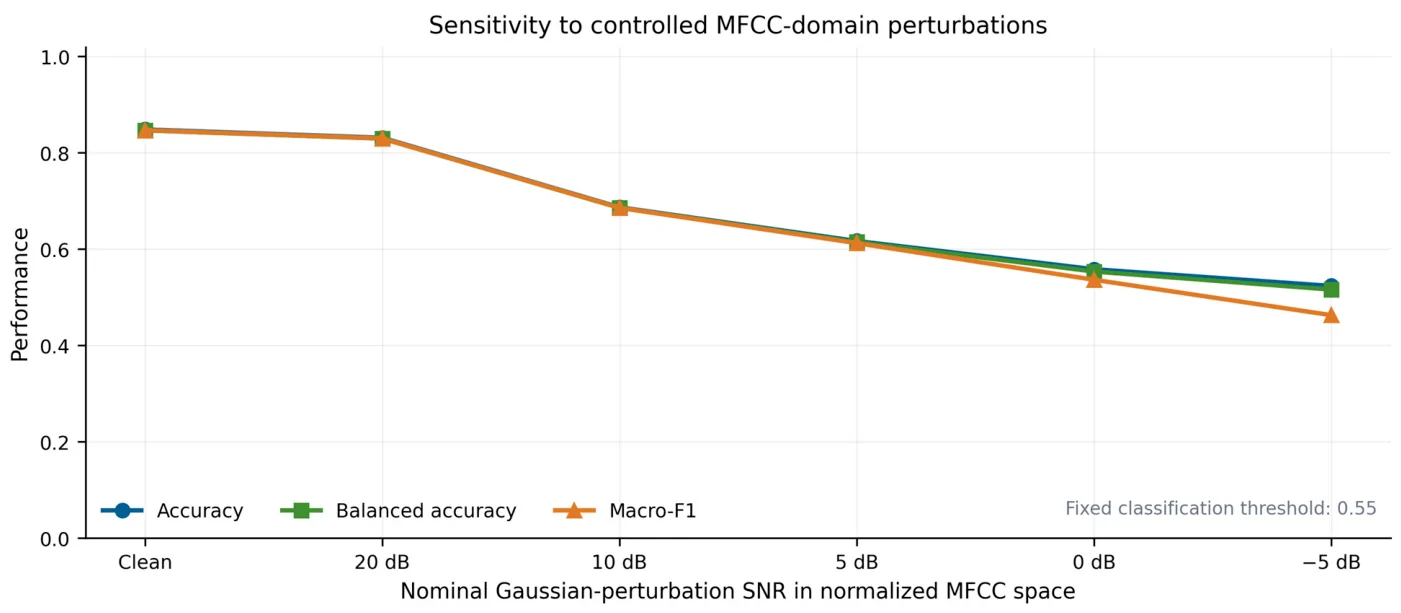
Original feature-domain Gaussian-perturbation visualization retained without alteration. This is distinct from the revised waveform-level stress test in Table 12 and is not used as evidence of physical-noise tolerance.

**Table 10:** Loose-state recall across archived propagation-distance categories, mean ± SD across five seeds.

Category	N	Full Recall	Labeled Recall	Interpretation
A	1065	0.9833 ± 0.0082	0.9816 ± 0.0067	Descriptive category
B	1930	0.9907 ± 0.0036	0.9850 ± 0.0133	Descriptive category
C	2153	0.9894 ± 0.0079	0.9844 ± 0.0113	Descriptive category
D	1253	0.9927 ± 0.0056	0.9898 ± 0.0058	Descriptive category
E	2462	0.9908 ± 0.0047	0.9811 ± 0.0166	Descriptive category
F	1540	0.9716 ± 0.0122	0.9536 ± 0.0441	Descriptive category

**Table 11:** Loose-state recall across archived loosened-bolt-count bins, mean ± SD across five seeds.

Category	N	Full Recall	Labeled Recall	Interpretation
1–4	649	0.9806 ± 0.0158	0.9837 ± 0.0048	Descriptive bin
5–9	5233	0.9845 ± 0.0067	0.9736 ± 0.0240	Descriptive bin
10–14	303	0.9967 ± 0.0040	0.9828 ± 0.0212	Descriptive bin
15–19	165	0.9988 ± 0.0027	0.9952 ± 0.0079	Descriptive bin
≥20	4053	0.9903 ± 0.0045	0.9856 ± 0.0066	Descriptive bin

**Table 12:** Waveform-level stress results after MFCC regeneration. Values are mean ± SD across five training seeds.

Waveform Condition	SNR dB	Full Macro-F1	Labeled Macro-F1	No Explicit Reconstruction
Regenerated clean	—	0.9114 ± 0.0033	0.9027 ± 0.0134	0.9068 ± 0.0043
Gaussian	20	0.4958 ± 0.0370	0.4936 ± 0.0517	0.5054 ± 0.0215
Gaussian	10	0.4829 ± 0.0394	0.4686 ± 0.0566	0.4541 ± 0.0829
Gaussian	0	0.3938 ± 0.1189	0.3810 ± 0.1008	0.3572 ± 0.1018
Recorded-background proxy	20	0.5355 ± 0.0407	0.5256 ± 0.0442	0.5186 ± 0.0506
Recorded-background proxy	10	0.4270 ± 0.0261	0.5002 ± 0.0685	0.3886 ± 0.0988
Recorded-background proxy	0	0.3143 ± 0.0657	0.3899 ± 0.0922	0.3280 ± 0.1025

### 3.7. Representation Evolution and Qualitative Reconstruction Behavior

As a descriptive representation diagnostic, 1400 class-balanced samples from the original analysis are used to trace silhouette and Fisher measures through the encoder. This visualization is not a matched ablation and does not establish the contribution of unlabeled reconstruction.

As shown in Table 13, the original MFCC has a silhouette coefficient and Fisher ratio of 0.0057, indicating substantial overlap between tight and loose samples in the input feature space. Both measures increase with encoder depth and reach 0.1825 and 0.4226, respectively, after the third conformer block. The shared encoder of the final model therefore progressively transforms the input into a representation that better separates the two labels in the sampled evaluation data. Because the same analysis was not performed for a matched model without unlabeled reconstruction, the layer-wise trend cannot be attributed specifically to the unlabeled pool. Figure 14 displays the layer-wise changes.

Qualitative visualizations illustrate the operation of the auxiliary objectives. The denoising head recovers patterns in Gaussian-corrupted MFCC arrays, and the masked-reconstruction head fills missing token-position and feature-coordinate regions using the remaining context. Figure 15 and Figure 16 show illustrative denoising and masked-reconstruction examples, respectively. These examples visually suggest plausible reconstruction in the implemented array and show the type of constraint supplied to the shared encoder; they are not interpreted as recovery of verified continuous time intervals or physical frequency bands. In the absence of a separate aggregate reconstruction-error analysis, the images are not used to quantify reconstruction fidelity. More importantly, successful-looking reconstruction does not itself demonstrate improved diagnosis; the closely matched comparison in Table 7 provides the relevant comparative evidence.

### 3.8. Illustrative Alert Burden

To illustrate the operational consequence of false positives, Table 14 combines the full model’s grouped-test loose recall and false-positive rate with assumed looseness prevalences. The calculation holds class-conditional behavior and the validation threshold fixed and refers to 1000 screened segments, not independent bolts or towers.

At 1% assumed prevalence, the measured rates imply approximately 9.9 true alerts and 205.5 false alerts per 1000 segments. This is an illustrative burden calculation rather than a prevalence estimate, field forecast, or deployment-threshold recommendation.

### 3.9. Computational Cost

At t = 1, the one-encoder shortcut produces exactly the same FP32 logits and binary decisions as the original two-encoder clean path across all 13,934 validation samples and five full-model checkpoints. The separately trained single-representation classifier reaches macro-F1 0.9098 ± 0.0087 versus 0.9124 ± 0.0033 for the duplicated-feature classifier; the paired interval [−0.0100, +0.0151] includes zero.

As shown in Table 15, at batch size 512, the one-encoder shortcut reduces mean batch time from 24.959 to 12.163 ms while preserving the original classifier. These timings exclude acquisition, preprocessing, file access, host-to-device transfer, and target-platform execution and therefore are not end-to-end field latency measurements.

## 4. Discussion

### 4.1. Role and Operating Regime of Unlabeled Reconstruction

The grouped five-seed analysis shows a small positive mean difference when unlabeled reconstruction is added to labeled reconstruction, but its 95% seed interval includes zero and only three of five pairs favor the full model. A conditional acquisition-group resampling interval is positive, but it conditions on the fitted models and inferred groups and cannot replace missing physical-tower identifiers. The masking-only unlabeled variant also slightly exceeds the full model in mean macro-F1, so the results support an auxiliary role for reconstruction without establishing that both objectives together are optimal.

All six reduced-label paired intervals include zero, and the mean differences change sign across budgets. The data therefore provide no general evidence that the present fixed reconstruction weights reduce annotation requirements. Component effects remain configuration dependent.

### 4.2. Engineering Implications of Condition-Wise Results

Held-out tower-type and later-cohort results are substantially weaker than the grouped within-archive test. The available identifiers cannot certify physical-tower independence, and only one later-cohort subgroup contains both classes. These findings limit the evidence to retrospective grouped performance rather than unseen-tower deployment.

Waveform-level Gaussian and recorded-background-proxy additions cause marked degradation, while low assumed prevalence produces many false alerts despite high loose recall. Together with unmeasured impact force, incomplete calibration records, and unavailable field-noise limits, these results require prospective tower-separated acquisition and field-specific threshold validation before operational use.

## 5. Conclusions

This study evaluates a reconstruction-assisted semi-supervised Conformer for transmission-tower bolt looseness diagnosis using an audited common cohort, grouped training/validation/test roles, and matched multi-seed runs. Labeled data define the diagnostic boundary, while labeled and unlabeled MFCCs support denoising and masked reconstruction without state pseudo-labels.

Across five seeds, the full model achieves grouped-test macro-F1 0.9124 ± 0.0033 and ROC–AUC 0.9692 ± 0.0025, compared with 0.9032 ± 0.0135 and 0.9644 ± 0.0074 for labeled reconstruction. The paired macro-F1 interval includes zero, the masking-only variant has a slightly higher mean, and no reduced-label budget shows a conclusive benefit. The evidence therefore supports useful within-archive classification but not a universal improvement from the complete unlabeled objective.

Held-out tower types, a later acquisition cohort, illustrative prevalence calculations, and waveform-level stress tests expose substantial generalization, false-alert, and noise-tolerance limitations. Clean inference can use one encoder call with exactly unchanged predictions. Prospective evaluation with certified physical-tower separation, documented excitation and calibration, representative disturbances, and target-specific thresholds remains necessary.

## Figures and Tables

**Figure 1:**
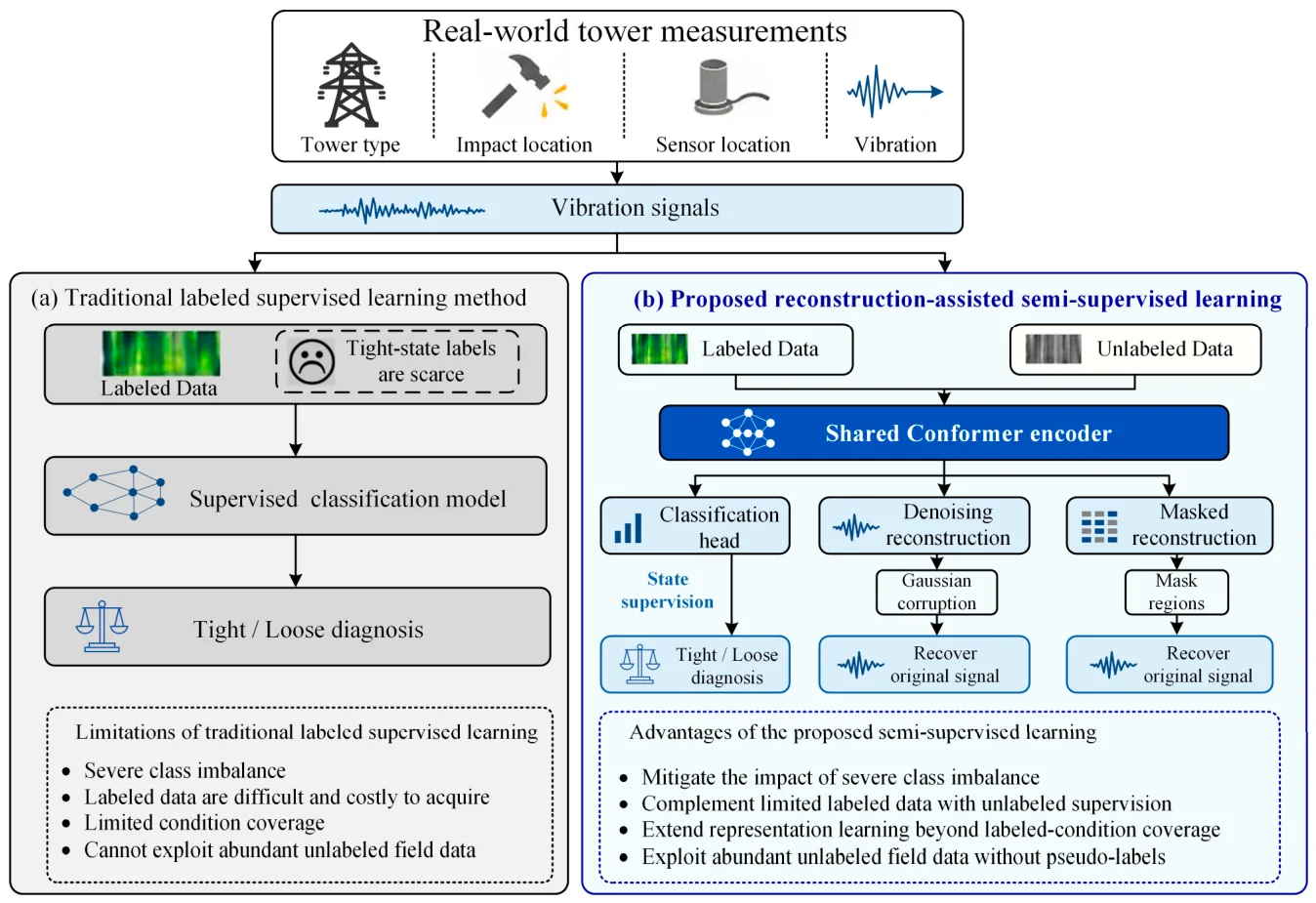
Comparison between traditional labeled supervised learning and the proposed reconstruction-assisted semi-supervised learning framework for transmission-tower bolt looseness diagnosis. The proposed method jointly exploits labeled and unlabeled MFCC data through classification and reconstruction objectives without assigning pseudo-labels to unlabeled samples.

**Figure 2:**
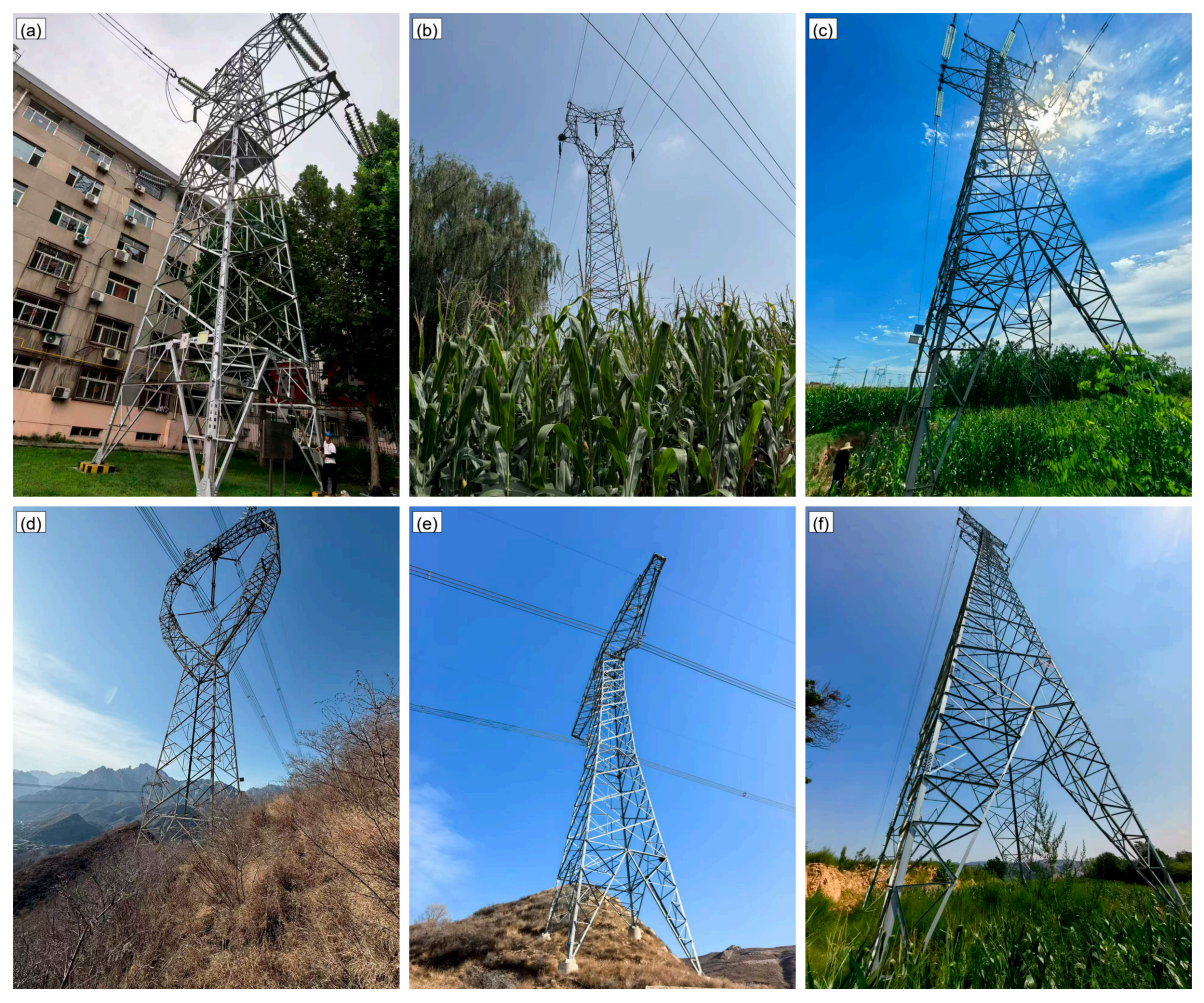
Representative field environments: (**a**) a lattice tower beside a multistory building and trees; (**b**) a tower surrounded by tall, cultivated vegetation; (**c**) a tower viewed from its base in agricultural land; (**d**) a tower on a vegetated hillside with mountainous terrain behind it; (**e**) a tower on sparsely vegetated, uneven terrain; and (**f**) a tower viewed from its base beside crops. The photographs illustrate visible surroundings and access conditions and are not used to infer voltage rating, physical tower identity, or experimental tower-type category.

**Figure 3:**
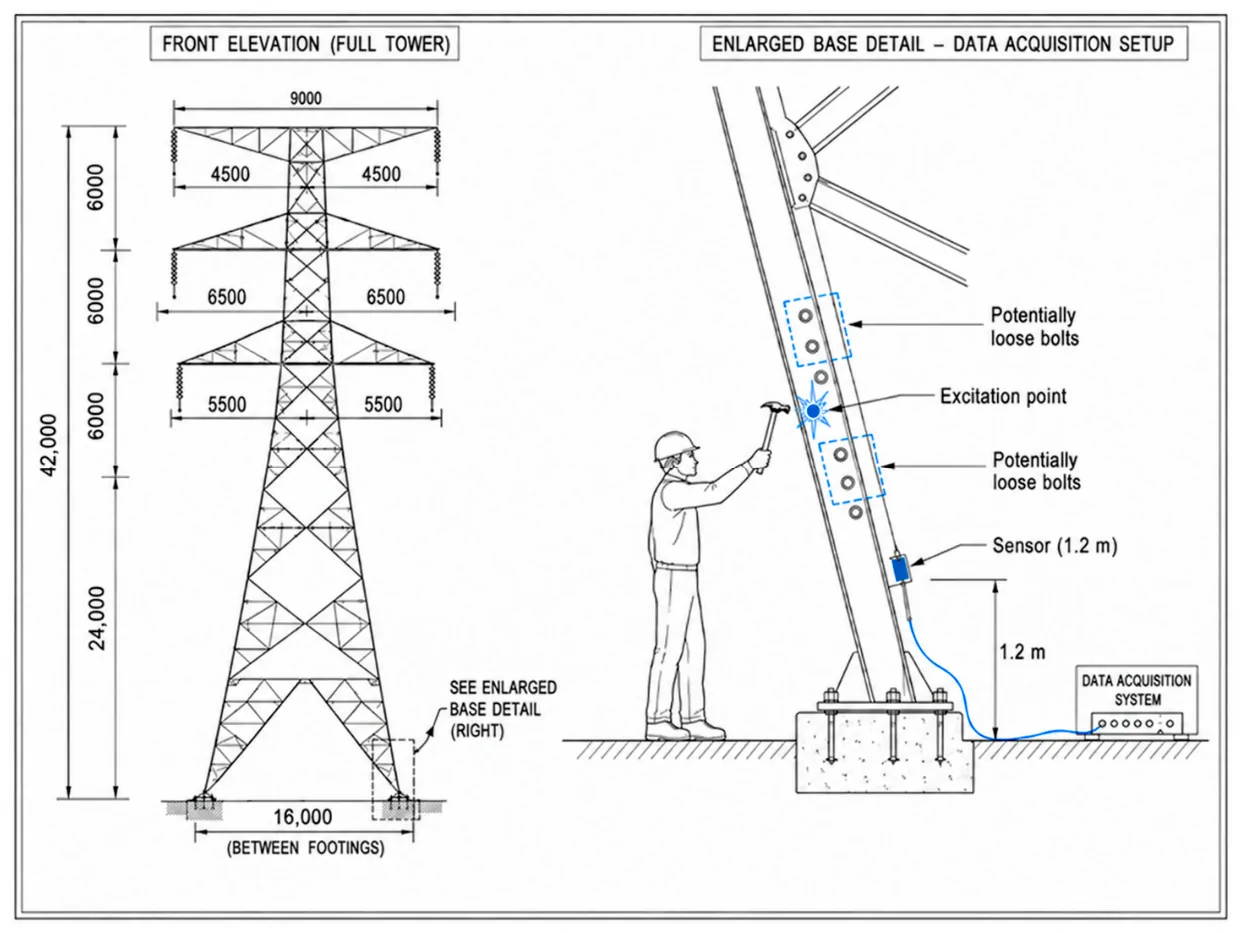
Schematic illustration of the transmission tower geometry and the locations of the excitation point, potentially loosened bolts, and sensor in the data acquisition setup.

**Figure 4:**
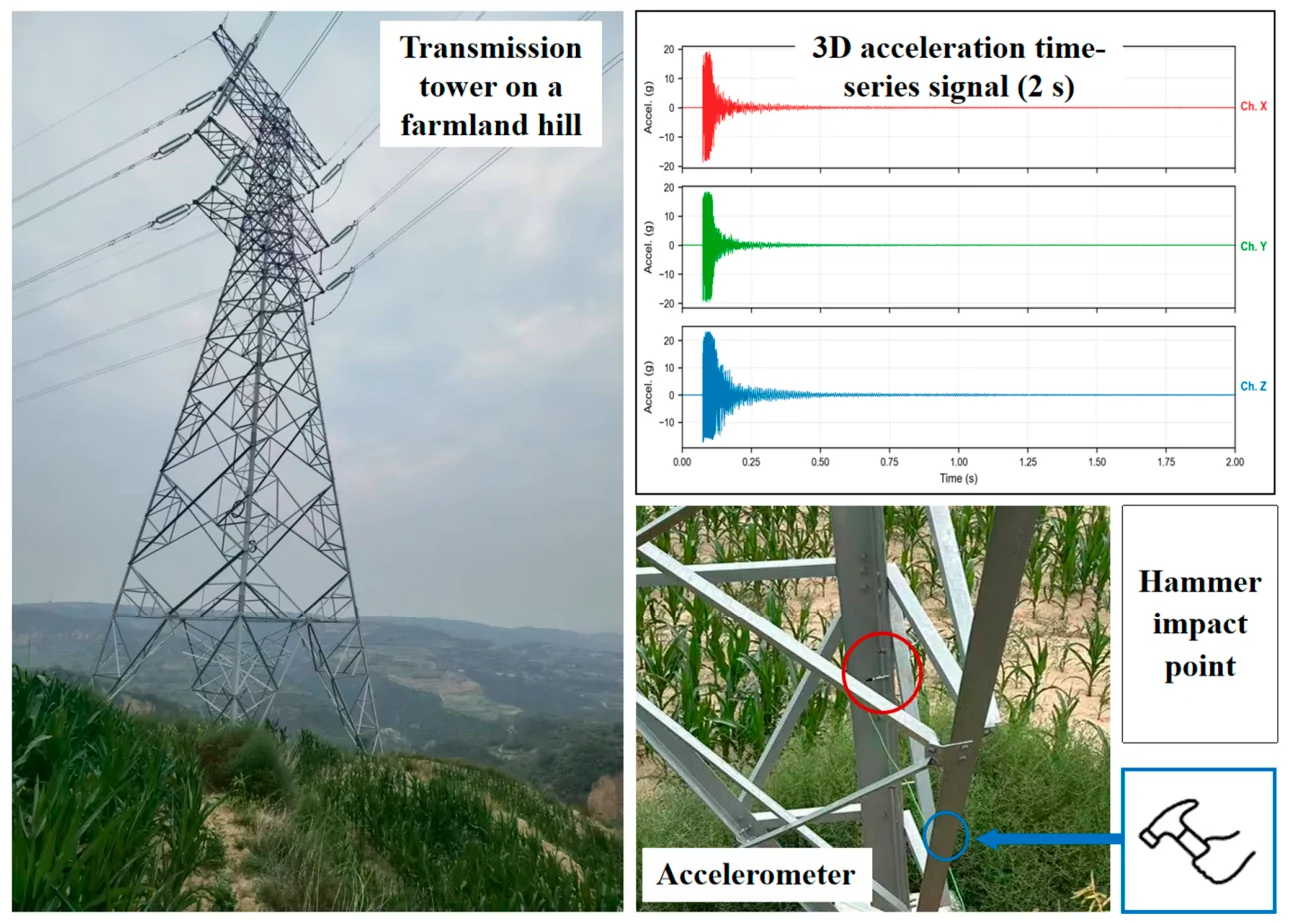
Field acquisition of hammer-excited vibration data on an in-service transmission tower.

**Figure 5:**
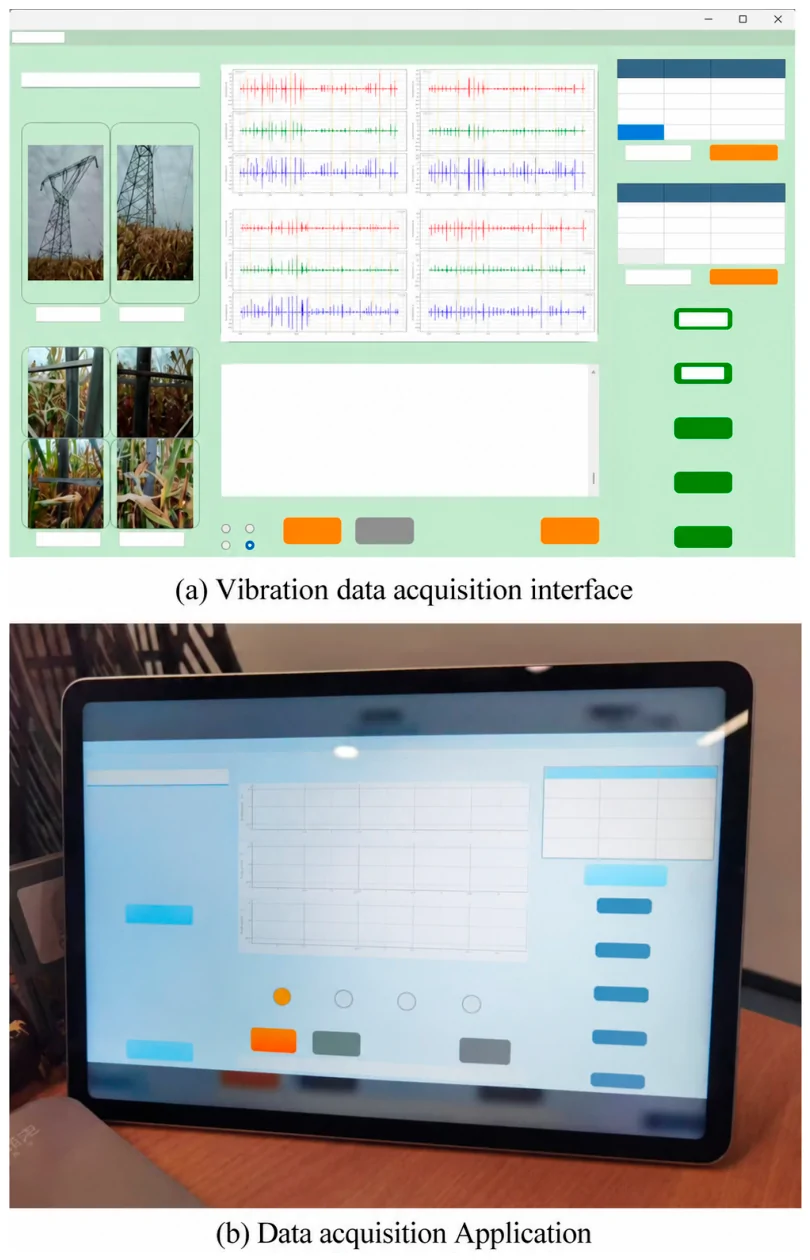
Graphical user interface of the tablet-based acquisition software. (**a**) Project initialization and tower selection screen with GIS-linked metadata fields; (**b**) real-time waveform monitoring panel showing tri-axial acceleration traces and the post-impact quality-control summary (peak acceleration, SNR estimate, waveform thumbnail). The interface was designed for use by field crews and features large touch-friendly controls and minimal text entry.

**Figure 7:**
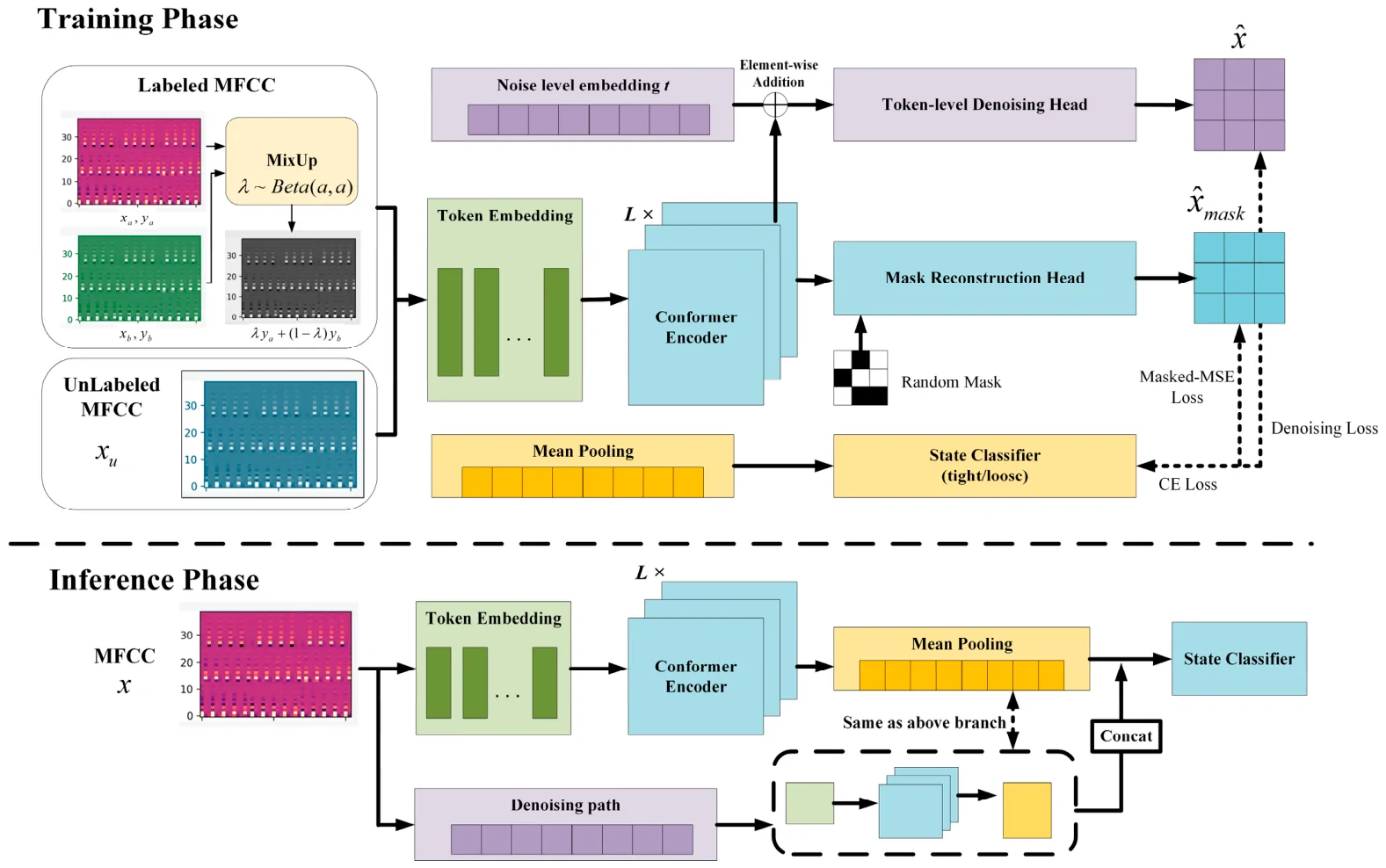
Original training and inference pathways of the conformer-denoising MFCC network. The training branches and classifier input are unchanged. At clean inference, the two identical encoder evaluations shown in the diagram are implemented with one encoder call followed by the same duplicated [h, h] classifier input.

**Figure 9:**
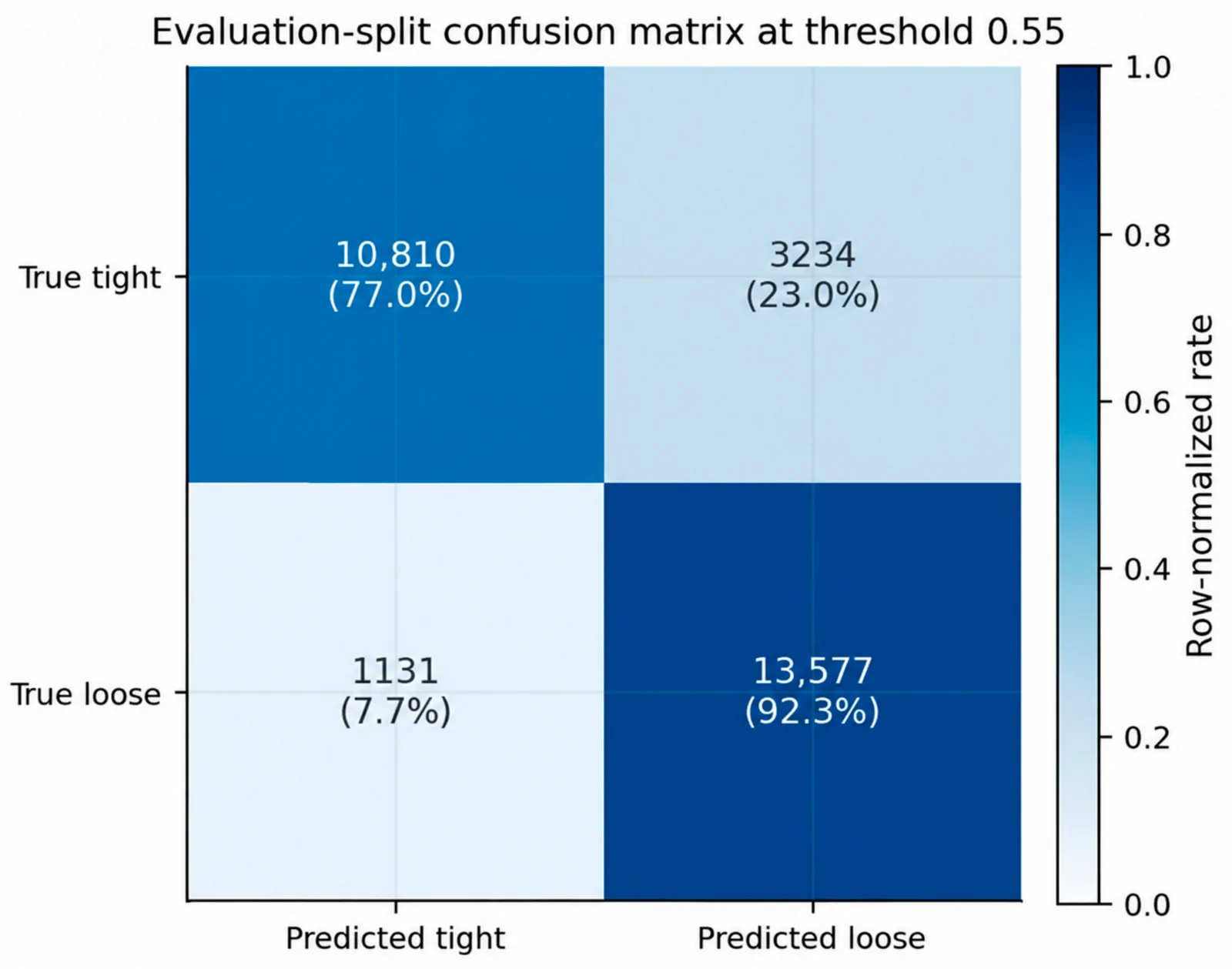
Original confusion matrix retained without alteration as a record of the initial internally calibrated analysis. Revised class-wise results under validation-only calibration are reported in Table 6.

**Figure 10:**
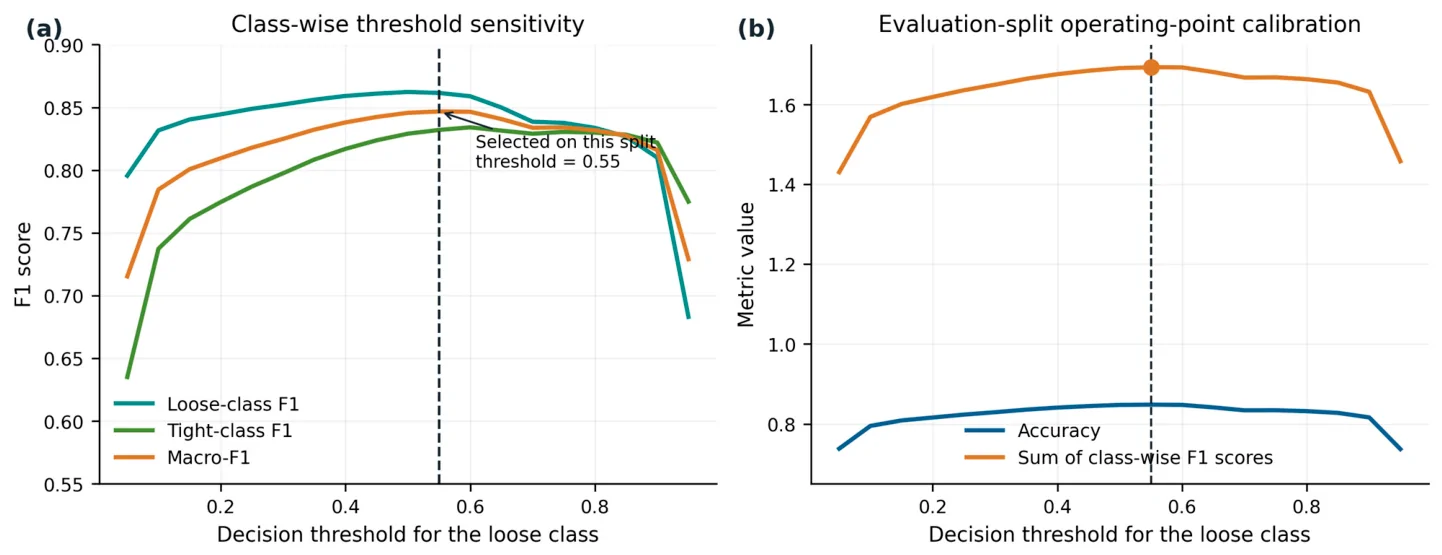
Original threshold-sensitivity visualization retained without alteration. The revised protocol does not select the operating point from this curve; all reported test thresholds are fixed from validation.

**Figure 11:**
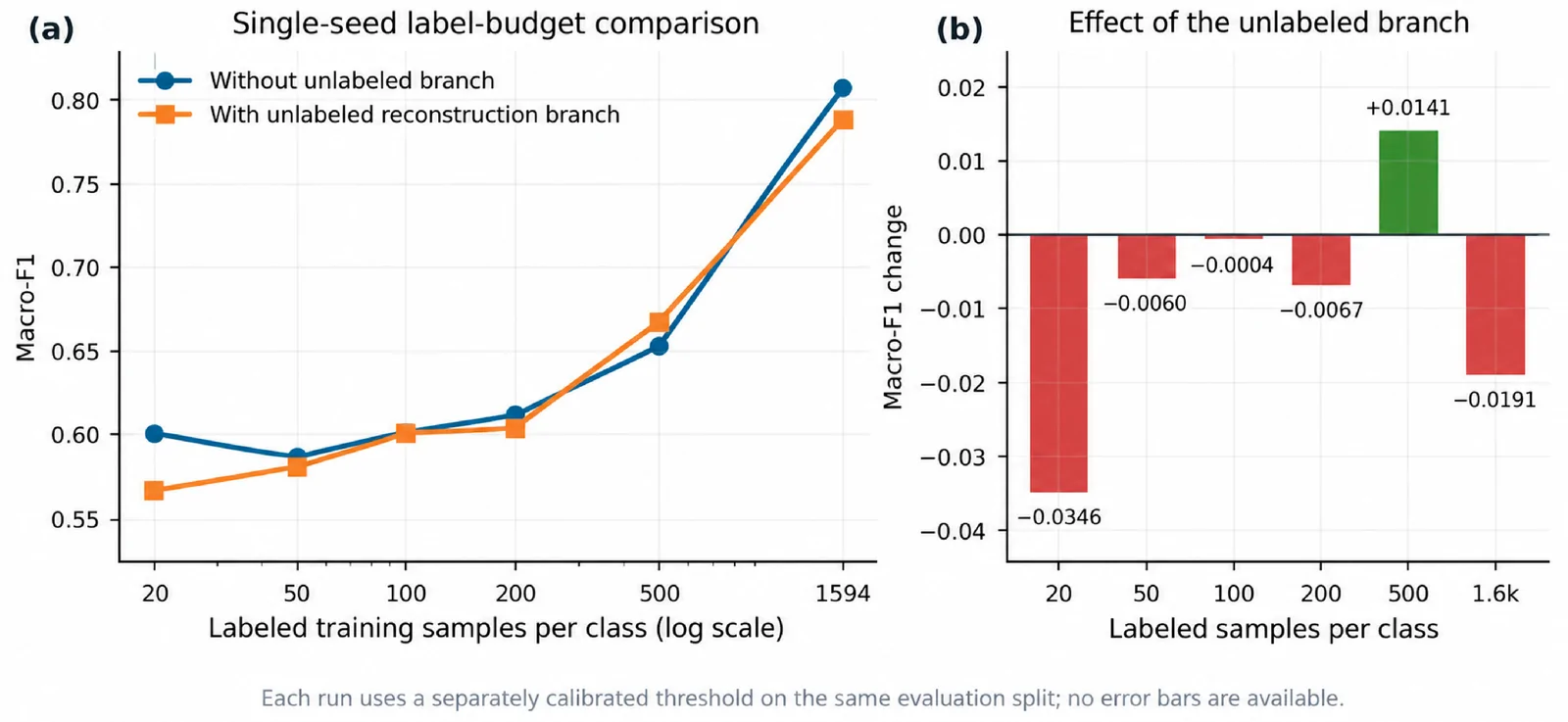
Original single-run label-budget visualization retained without alteration as an illustrative trajectory. Statistical interpretation is based on the paired five-seed results in Table 8.

**Figure 14:**
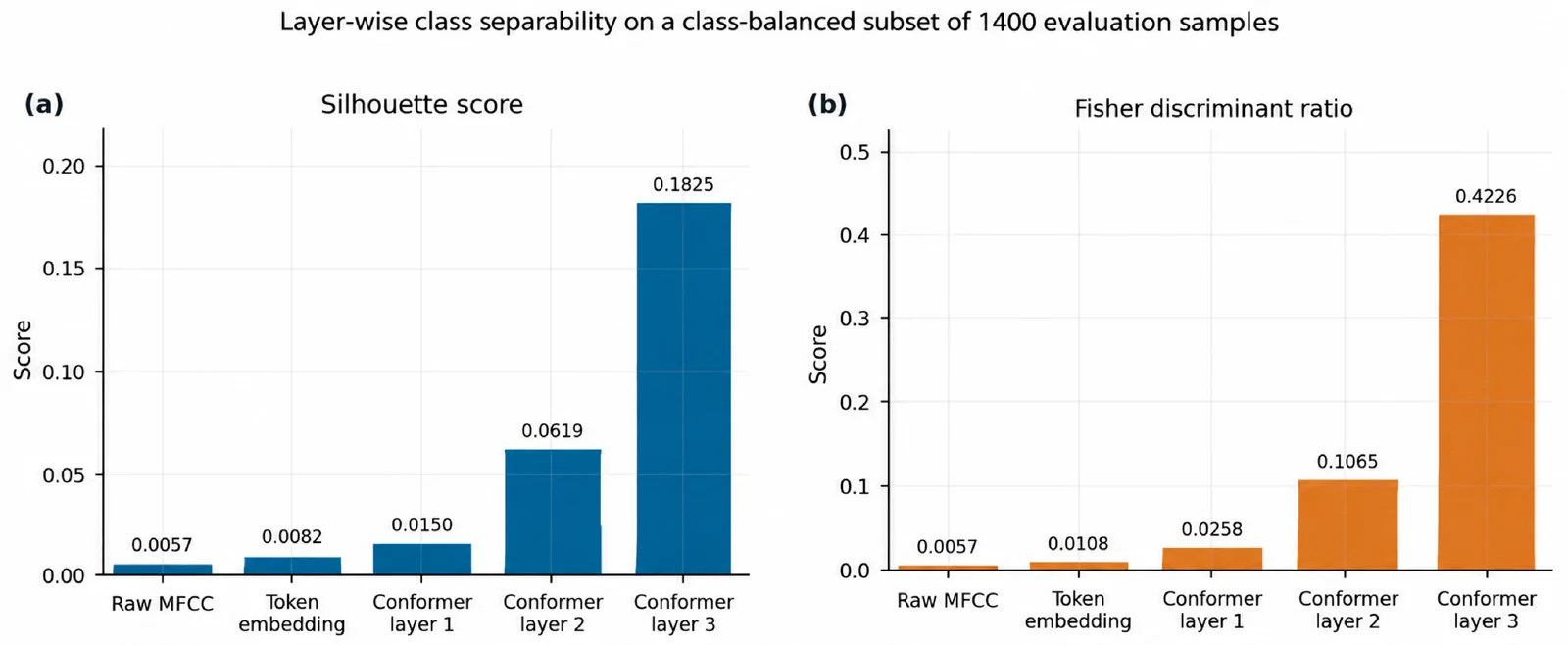
Original class-separability visualization retained without alteration as a descriptive within-model diagnostic; it is not used for the revised component-wise inference.

**Figure 15:**
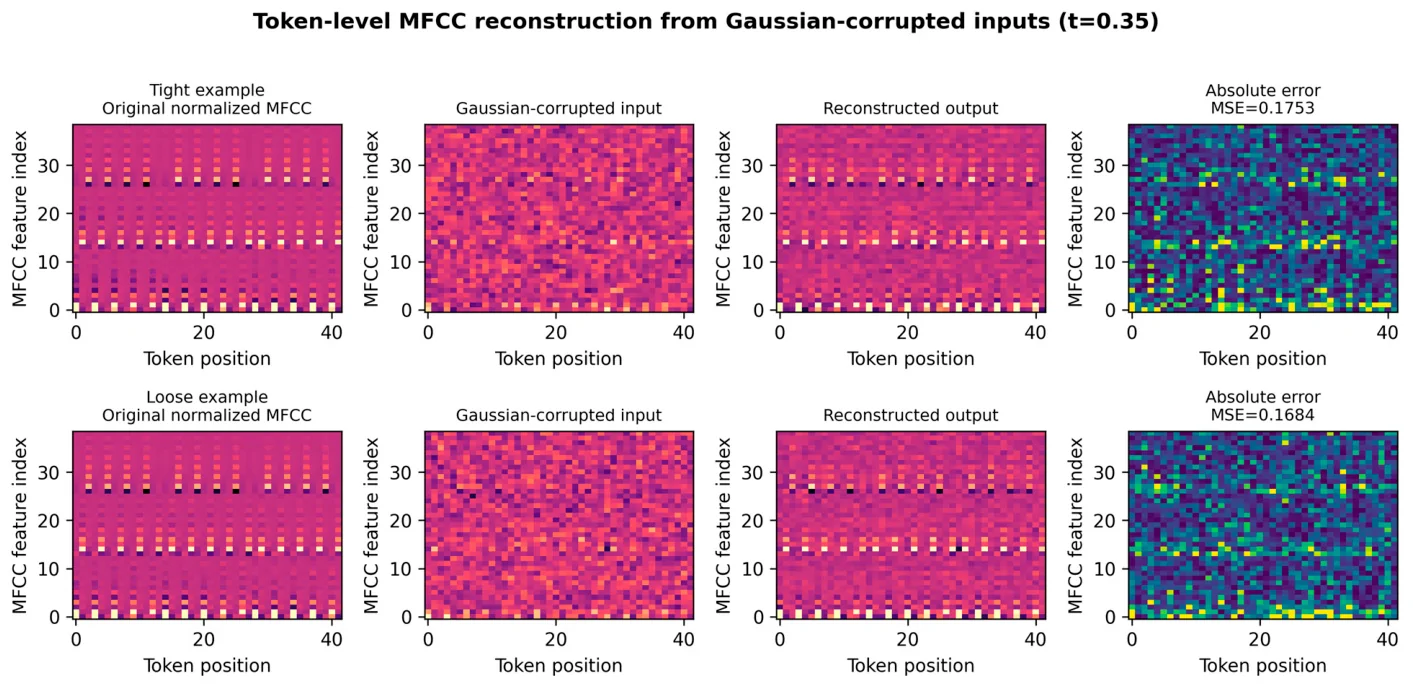
Illustrative token-level reconstruction from Gaussian-corrupted MFCC inputs at *t* = 0.35. The panels show the original normalized MFCC, corrupted input, reconstructed output, and absolute error. This visualization confirms the operation of the denoising objective but does not isolate the effect of unlabeled training data.

**Figure 16:**
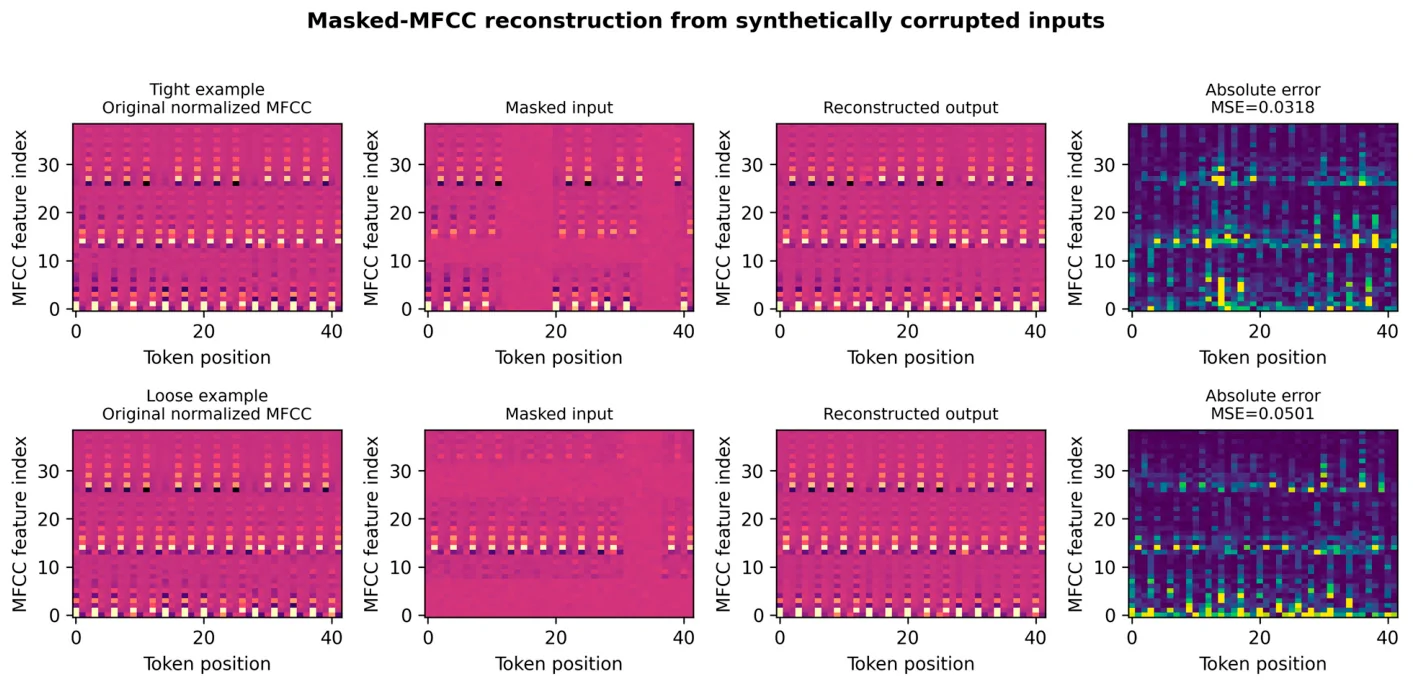
Illustrative masked-MFCC reconstruction for one tight and one loose sample. Contiguous token-position and feature-coordinate blocks are masked together with the small perturbation used during training. This visualization verifies reconstruction behavior but does not independently establish a classification gain from unlabeled data.

**Table 1:** Experimental setup and computational environment.

Module	Parameter/Specification
Excitation system	Handheld impact hammer with custom convex steel tip; no force transducer
Vibration sensor	STMicroelectronics IIS3DWB tri-axial digital MEMS vibration sensor; full-scale setting ±16 g; signal bandwidth approximately 6 kHz (−3 dB); sensitivity 0.488 mg/LSB; output data rate 26,667 Hz; magnetic mounting base
Data acquisition unit	Custom digital acquisition unit for synchronous tri-axial IIS3DWB output; USB interface to field tablet
Field terminal	Rugged tablet computer running a custom acquisition application

**Table 3:** Layered model architecture and principal control settings. Dimensions refer to one sample unless stated otherwise.

Stage	Output	Settings
MFCC input	42 × 39	Three axis blocks of 14 frames; static, delta and delta–delta coefficients
Embedding and position	42 × 160	Linear 39→160; LayerNorm; dropout 0.15; learned 42 × 160 position array
Conformer block 1	42 × 160	Two half-step FFNs, width 640; four attention heads; convolution kernel 7
Conformer block 2	42 × 160	Same dimensions and settings as block 1
Conformer block 3	42 × 160	Same dimensions and settings as block 1
Mean pooling	160	Average over 42 positions
Denoising head	42 × 39	LayerNorm 160; Linear 160→320; GELU; dropout; Linear 320→39
Condition embeddings	160 each	Time: 1→160→160 with SiLU; label: three entries including null
Masked head	42 × 39	Independent head with same dimensions as denoising head
Full classifier	2 logits	LayerNorm 320; Linear 320→160→80→2; GELU and dropout
Single control	2 logits	Classifier input 160; remaining classifier widths 160→80→2
Transformer control	42 × 160	Three pre-LN blocks; four heads; FFN width 640; same heads and losses
Raw CNN	2 logits	Channels 32/64/128/256; kernels 33/17/9/9; stride 4; BN/ReLU; pool; 64→2 head
MFCC LSTM	2 logits	Two bidirectional layers, hidden 128; dropout 0.2; last state; LN 256; 128→2 head
VMD MLP	2 logits	50→1500→800→500→2; ReLU; dropout 0.2

**Table 4:** Frozen training, augmentation, loss, and validation-selection settings used in the revised experiments.

Setting	Value
Optimizer and schedule	AdamW; learning rate 0.0003; weight decay 0.0001; cosine decay; gradient norm clip 1; mixed precision
Epochs and batch	30 epochs for complete-data/type-transfer runs; 80 for label budgets; labeled/unlabeled batch 512
Seeds	17, 29, 43, 71, 101; type-transfer uses 17, 29, 43
Class sampling	Equal class probability with replacement; epoch draws equal training subset size
MixUp and smoothing	Beta(0.2, 0.2); label smoothing 0.03
Clean-view masking	Probability 0.2; one width 1–4 interval per array dimension
Reconstruction masking	Two width 1–8 intervals per dimension; Gaussian standard deviation 0.05
Denoising coefficient	sigmoid of N(−0.8, 0.8^2^), minimum 0.05; unit Gaussian corruption
Null label condition	Probability 0.1 for labeled denoising; always null for unlabeled denoising
Loss weights	Clean CE 1.5; noisy CE 0.5; labeled denoising 0.1; labeled masking 0.03; unlabeled denoising/masking 0.02 each
Checkpoint	Earliest maximum validation macro-F1 at threshold 0.5, evaluated each epoch
Calibration and threshold	Validation NLL temperature fit, T ∈ [0.1, 10]; validation macro-F1 threshold 0.05:0.05:0.95, smallest tie

**Table 9:** Leave-one-recorded-tower-type-out results, mean ± SD across three seeds. A dash indicates a single-class cohort.

Held-Out Type	Segments	Full Macro-F1	Labeled Macro-F1	Full Specificity
220 kV type 4	9644	0.4964 ± 0.0549	0.5164 ± 0.0370	0.0671 ± 0.0745
220 kV type 5	880	—	—	0.0420 ± 0.0366
500 kV type 1	25,807	0.3309 ± 0.0152	0.3345 ± 0.0159	0.6850 ± 0.0438
500 kV type 2	18,246	0.4464 ± 0.0350	0.4546 ± 0.0180	0.1392 ± 0.0699
500 kV type 3	15,832	0.4825 ± 0.0078	0.4902 ± 0.0132	0.0176 ± 0.0074

**Table 13:** Class separability at successive representation stages.

Representation Stage	Silhouette Coefficient	Fisher Discriminant Ratio
Original MFCC	0.0057	0.0057
Token embedding	0.0082	0.0108
Conformer block 1	0.0150	0.0258
Conformer block 2	0.0619	0.1065
Conformer block 3	0.1825	0.4226

**Table 14:** Illustrative alert burden under assumed looseness prevalence. Values are means of five seed-specific calculations.

Assumed Prevalence	True Alerts	False Alerts	Missed Loose Segments	Positive Predictive Value
1%	9.9	205.5	0.1	0.046
5%	49.4	197.2	0.6	0.201
10%	98.7	186.9	1.3	0.346
20%	197.4	166.1	2.6	0.544

**Table 15:** GPU forward-pass timing on an otherwise idle RTX 3090. Values are mean ± SD across five checkpoints, in milliseconds per batch.

Batch Size	Original Forward	Two Clean Encoder Calls	One Encoder Shortcut
1	3.914 ± 0.049	3.463 ± 0.062	1.837 ± 0.032
128	6.950 ± 0.056	6.740 ± 0.049	3.376 ± 0.030
512	24.959 ± 0.207	24.261 ± 0.177	12.163 ± 0.094

## Data Availability

The accompanying supplementary archive contains frozen analysis code and configurations, sample and partition manifests, validation outputs, test and external predictions, and statistical result files. Raw waveforms, MFCC feature caches, and trained checkpoint weights are not included; requests for unavailable data should be directed to the corresponding author.

## Supplementary Materials

The following supporting information can be downloaded at: https://www.mdpi.com/article/10.3390/s26195998/s1, Video S1: Data acquisition procedure.

## References

[1] Huang J., Liu J., Gong H., Deng X. (2022). A comprehensive review of loosening detection methods for threaded fasteners. Mech. Syst. Signal Process..

[2] Wu J., Shao J., Zhou G., Yang D., Cheng Y. (2021). Detection of bolt looseness based on average autocorrelation function. Shock Vib..

[3] Yang W., Zhang Z., Chen X. (2023). Vibration-based looseness identification of bolted structures via quasi-analytic wavelet packet and optimized large margin distribution machine. Struct. Health Monit..

[4] Wang F. (2023). Multi-bolt looseness detection using a new acoustic emission strategy. Struct. Health Monit..

[5] Wang X., Yue Q., Liu X. (2024). Bolted lap joint loosening monitoring and damage identification based on acoustic emission and machine learning. Mech. Syst. Signal Process..

[6] Jiang J., Chen Y., Dai J., Liang Y. (2022). Multi-bolt looseness state monitoring using the recursive analytic based active sensing technique. Measurement.

[7] Na W.S. (2022). A portable bolt-loosening detection system with piezoelectric-based nondestructive method and artificial neural networks. Struct. Health Monit..

[8] Ma T., Feng Q., Tan Z., Ou J. (2024). Optical phase mode analysis method for pipeline bolt looseness identification using distributed optical fiber acoustic sensing. Struct. Health Monit..

[9] Tong T., Hua J., Du D., Gao F., Lin J. (2025). Bolt looseness detection in lap joint based on phase change of Lamb waves. Mech. Syst. Signal Process..

[10] Jiang Y.-Z., Zhang Y.-B., Zeng L.-Y., Huang L., Su B.-W., Chen L.-C. (2026). A new magnetic method for monitoring the tightness of bolted flanged connection. Measurement.

[11] Zhao L., Liu Z., Yuan P., Wen G., Huang X. (2024). Vibration feature extraction and fault detection method for transmission towers. IET Sci. Meas. Technol..

[12] Liu Z., Huang X., Zhao L., Wen G., Feng G., Zhang Y. (2023). Research on online monitoring technology for transmission tower bolt looseness. Measurement.

[13] E X., Wang W. (2023). Escalator foundation bolt loosening fault recognition based on empirical wavelet transform and multi-scale gray-gradient co-occurrence matrix. Sensors.

[14] Li Q., Li M., Zhu B., Hu X. (2023). Research on Anti-loosening Technology of Transmission Line Bolts. MATEC Web. Conf..

[15] Jiang W., Mo Z., Wang T., Zhang Q., Yang L., Zhang W. (2025). Loosening process of bolted connection in transmission tower under transverse cyclic load. Structures.

[16] Liu H., He P., Lu Z., Li J., Lu Z. (2025). Study on monitoring the loose bolts of transmission tower by vibration signal. J. Vibroengineering.

[17] Wang F., Song G. (2021). 1D-TICapsNet: An audio signal processing algorithm for bolt early looseness detection. Struct. Health Monit..

[18] Wang F., Song G. (2021). A novel percussion-based method for multi-bolt looseness detection using one-dimensional memory augmented convolutional long short-term memory networks. Mech. Syst. Signal Process..

[19] Han Q., Pan Y., Yang D., Xu Y. (2022). CNN-based bolt loosening identification framework for prefabricated large-span spatial structures. J. Civ. Struct. Health Monit..

[20] Cao J., Xue R., Du S. (2026). Learning multi-pattern vibration representations for in-the-wild bolt looseness detection on power transmission towers. J. Eng..

[21] Zhao L., Wen G., Wang J., Liu Z., Huang X. (2025). Transmission tower bolt-loosening time-frequency analysis and localization method considering time-varying characteristics. Struct. Health Monit..

[22] He P., Lu Z., Liu H., Li J., Lu Z. (2025). Research on voice print recognition of loose state of transmission tower bolts based on MFCC feature vector. Noise Vib. Worldw..

[23] Qin Y., Yuan L. (2025). Intelligent bolt loosening detection in transmission towers using acoustic signature analysis and machine learning. Processes.

[24] Liu P., Wang X., Wang Y., Zhu J., Ji X. (2024). Research on percussion-based bolt looseness monitoring under noise interference and insufficient samples. Mech. Syst. Signal Process..

[25] Zhou L., Chen S.X., Ni Y.Q., Choy A.W.H. (2021). EMI-GCN: A hybrid model for real-time monitoring of multiple bolt looseness using electromechanical impedance and graph convolutional networks. Smart Mater. Struct..

[26] Du F., Wu S., Xu C., Yang Z., Su Z. (2023). Electromechanical impedance temperature compensation and bolt loosening monitoring based on modified UNet and multitask learning. IEEE Sens. J..

[27] Nguyen T.-T., Ta Q.-B., Ho D.-D., Kim J.-T., Huynh T.-C. (2023). A method for automated bolt-loosening monitoring and assessment using impedance technique and deep learning. Dev. Built Environ..

[28] Du C., Liu J., Gong H., Huang J., Zhang W. (2024). Percussion-based loosening detection method for multi-bolt structure using convolutional neural network DenseNet-CBAM. Struct. Health Monit..

[29] Chen Y., Zhu L., Gao Z., Li W., Wu J. (2025). Multi-bolt looseness positioning using multivariate recurrence analytic active sensing method and MHAMCNN model. Struct. Health Monit..

[30] Chen X., Zhou W., Yang J., Zhang X., He C. (2025). A percussion method with attention-based sequence modeling for bolt looseness detection and adaptive anomaly recognition. Measurement.

[31] Furusawa T., Premachandra C., Kihara S., Takizawa M., Katsuragi H., Hashimura S., Hosoya N. (2025). AI-based bolt/nut looseness sensing using spectrogram images of shaft vibrations. IEEE Sens. J..

[32] Hu M., Yue N., Groves R.M. (2024). Damage classification of a bolted connection using guided waves and explainable artificial intelligence. Procedia Struct. Integr..

[33] Ramasso E., de Oliveira Teloli R., Marcel R. (2025). On the condition monitoring of bolted joints through acoustic emission and deep transfer learning: Generalization, ordinal loss, and super-convergence. Struct. Health Monit..

[34] Pérez-Pérez E.-J., Puig V., López-Estrada F.-R., Valencia-Palomo G., Santos-Ruiz I., Samada S.E. (2023). Fault detection and isolation in wind turbines based on neuro-fuzzy qLPV zonotopic observers. Mech. Syst. Signal Process..

[35] He D., Xu Y., Sun H., Jin Z., Zhao M. (2025). Self-supervised learning for vehicle bearing fault diagnosis based on time-frequency dual-domain contrast and fusion. Nonlinear Dyn..

[36] Xu Y., He D., Sun H., Jin Z., Zhao M. (2026). Self-supervised learning for train bearing fault diagnosis based on time-frequency dual domain prediction. Struct. Health Monit..

[37] Hu X., Jiang C., Huang Y., Peng D., Su H., He Y., Chen Z. (2026). SMNet: A novel compositional generalization model for industrial robot multi-joint fault diagnosis. IEEE Internet Things J..

[38] Hu X., Zhang H., Cao J., Huang Y., Zhang X., Wang H., Tang T. (2026). PSRONet: A deep reinforcement learning-based sensor configuration framework in railway point machines fault diagnosis. IEEE Trans. Instrum. Meas..

[39] Sun W., Guan Z., Zeng Y., Pan J., Gao Z. (2022). Bolt loosening detection of rocket connection structure based on variational modal decomposition and support vector machines. Appl. Sci..

[40] Kim D.-Y., Kim M.-J., Kim C.-I., Yoon G.H. (2025). Looseness detection system of bolted joints using a VMD-based nonlinear transformation approach with deep residual network. Meas. Sci. Technol..

